# Development of image analysis software for quantification of viable cells in microchips

**DOI:** 10.1371/journal.pone.0193605

**Published:** 2018-03-01

**Authors:** Maximilian Georg, Tamara Fernández-Cabada, Natalia Bourguignon, Paola Karp, Ana B. Peñaherrera, Gustavo Helguera, Betiana Lerner, Maximiliano S. Pérez, Roland Mertelsmann

**Affiliations:** 1 Department of Hematology and Oncology, University of Freiburg Medical Center, Freiburg, Germany; 2 National Technological University (UTN), Regional Faculty from Haedo, Paris, Buenos Aires, Argentina; 3 Faculty of Engineering - Institute of Biomedical Engineering - University of Buenos Aires (UBA), Buenos Aires C1063ACV, Argentina; 4 Biology and Experimental Medicine Institute (IBYME CONICET), Buenos Aires C1428ADN, Argentina; Hungarian Academy of Sciences, HUNGARY

## Abstract

Over the past few years, image analysis has emerged as a powerful tool for analyzing various cell biology parameters in an unprecedented and highly specific manner. The amount of data that is generated requires automated methods for the processing and analysis of all the resulting information. The software available so far are suitable for the processing of fluorescence and phase contrast images, but often do not provide good results from transmission light microscopy images, due to the intrinsic variation of the acquisition of images technique itself (adjustment of brightness / contrast, for instance) and the variability between image acquisition introduced by operators / equipment. In this contribution, it has been presented an image processing software, Python based image analysis for cell growth (PIACG), that is able to calculate the total area of the well occupied by cells with fusiform and rounded morphology in response to different concentrations of fetal bovine serum in microfluidic chips, from microscopy images in transmission light, in a highly efficient way.

## Introduction

Since the beginning of cell biology, scientists have sought methods to isolate and cultivate different cell lines for the investigation of cell and dynamics biology and their subsequent clinical application [1]. In cell cultures, special combinations of nutrients are required in the culture media to provide optimum conditions for the survival and in vitro growth of the different cell lines under study [2].

To maintain cell function and allow cell division and proliferation, the culture medium is universally complemented with fetal bovine serum (FBS), a mixture containing growth factors among its components [3]. FBS was constituted as a standard supplement of the cell culture medium, which is easily obtained and contains a high concentration of growth factors and a low concentration of gammaglobulins, compared to other sera originated from animals [4]. Normally, FBS is used to supplement the culture medium at a concentration of 5% to 20%. Only about 200 of the thousand of components that are present in the FBS composition have been defined. These components include hormones, vitamins, nucleosides, amino acids, lipids, carrier proteins (albumin, globin and transferrin), extracellular matrix components (fibronectin and laminin), stabilizing factors, detoxifying agents, proliferation factors and growth factors [5]. Many components of the culture medium can affect the rate of cell proliferation, but serum represents one of the best documented modulators of cell division and growth [6].

Microfluidics allows the miniaturization of conventional operations that occur in a conventional biological or chemical laboratory. Microfluidics applied to cell culture, as compared to static culture, is not only capable of maintaining well-defined cell culture conditions, also enables cells to be continuously supplied with oxygen, carbon dioxide and nutrients whereas the metabolic products are removed at a controlled rate [7], [8],[9]. Lab-on-a-chip technology has been widely accepted by biological and medical scientific communities as a promising tool for the control of the microenvironment at the molecular, cellular and tissue levels [7].

Due to the large amount of data that results from microfluidic chips, it is necessary to develop new tools that allow the analysis of images with powerful processors and algorithms. This combination of advanced image analysis and computation has assisted the modern biologist to observe dynamic phenomena and quantify the processes involved. Therefore, image analysis is a main objective within biology and requires intuitive software packages that facilitate image processing and with which the greatest possible amount of data is obtained quickly [10].

There are many options for open access image analysis, originally developed to solve the needs of particular cases that were subsequently extended for other purposes, such as ImageJ [11], BioImageXD [12], Icy [13], Fiji [14], Vaa3D [15], CellProfiler [16], 3D Slicer [17], Image Slicer [18], Reconstruct [19], FluoRender [20], ImageSurfer [21], OsiriX [22], and IMOD [23] among others [24].

There are also several analytical tools already on the market. However, many of them, despite being useful for the processing of fluorescence and phase contrast images, often do not provide good results from transmission light microscopy images, due to the intrinsic variation of the acquisition technique itself and the variability introduced between image acquisition by operators and by own equipment [25].

In this work, software has been developed: Python based image analysis for cell growth (PIACG), which allows automatic and high precision processing of images obtained during the experimental phase, providing in a quick and simple way a multitude of statistical data. As a proof of concept to test the developed software, the effects of different serum concentrations on the proliferation and replicative life span of cultured HEK 293T cell line have been analyzed.

To test the software, image acquisition was performed by adjusting the parameters for capturing images in transmission light microscopy with optimum quality for automatic processing and by designing of robustly fast image processing algorithms. The developed software is applied to the analysis of 1000 images of HEK-293T cells for 10 days. Software accuracy has been evaluated by comparing the cellular areas identified by the software with respect to manual counts. The results of the observed cellular growth and the numerical and statistical analysis of the software have also been compared. The software is able to segment and quantify images with diverse cellular densities and different cellular morphology automatically, without parameter adjustment.

## Material and methods

### Software development

In order to perform the image analysis of the cells cultured in microdevices, it is required a software package that could analyze large number of cells attached to uneven surfaces, with subtle gradients in background hues and at the same time that could discriminate between objects that are not cells such as chamber boundaries of the microfluidic channels. The image analysis software developed in this work that allows to deal with these issues was created using Python, for which there are many image processing libraries available. The following libraries were used: Mahotas [26], Matplotlib [27] and NumPy [28].

In perfectly focused images of cells under a light microscope, the membrane is a well visible, dark line while everything inside the cell has nearly the same shade as the area that surrounds it. For the purpose, it was necessary that the whole cell stands out from the background. Therefore, the focus was increased slightly further: the cells became slightly blurred but constantly darker than the background.

PIACG cuts every image in several pieces (36 by default) and processes each slice individually. This is required because of the uneven chamber surface: the irregularities cast shadows so that the background has many differently shaded areas. For precise results, adquisition of images that are as homogeneous as possible is essential.

During processing, the Mahotas library is used to generate an optimal threshold value and a Gaussian filter is applied to each slice. For the threshold, the Ridler-Calvard method was used. Then the generated threshold is employed to the gauss-version of the image. The sigma parameter was used. As the value for sigma in the Gaussian filter, 6 pixels were used. The result is an image in which all pixels that belong to cells are black and everything else is white.

In the last step the black pixels are counted and compared to the images total number of pixels. These values can be used to calculate the area that is covered by cells in percentage. If the pixels per inch or centimeter are known, the value can be calculated in square millimeter or any other requested unit.

To compare the functionalities of PIACG, the following steps were performed on ImageJ in order to analyze the same images: Open image / Selection of the area to quantify / Edit>Clear outside / Process>Binary>Convert to mask / Analyze>Measure.

### Microfluidic devices design and fabrication

**Fig 1:**
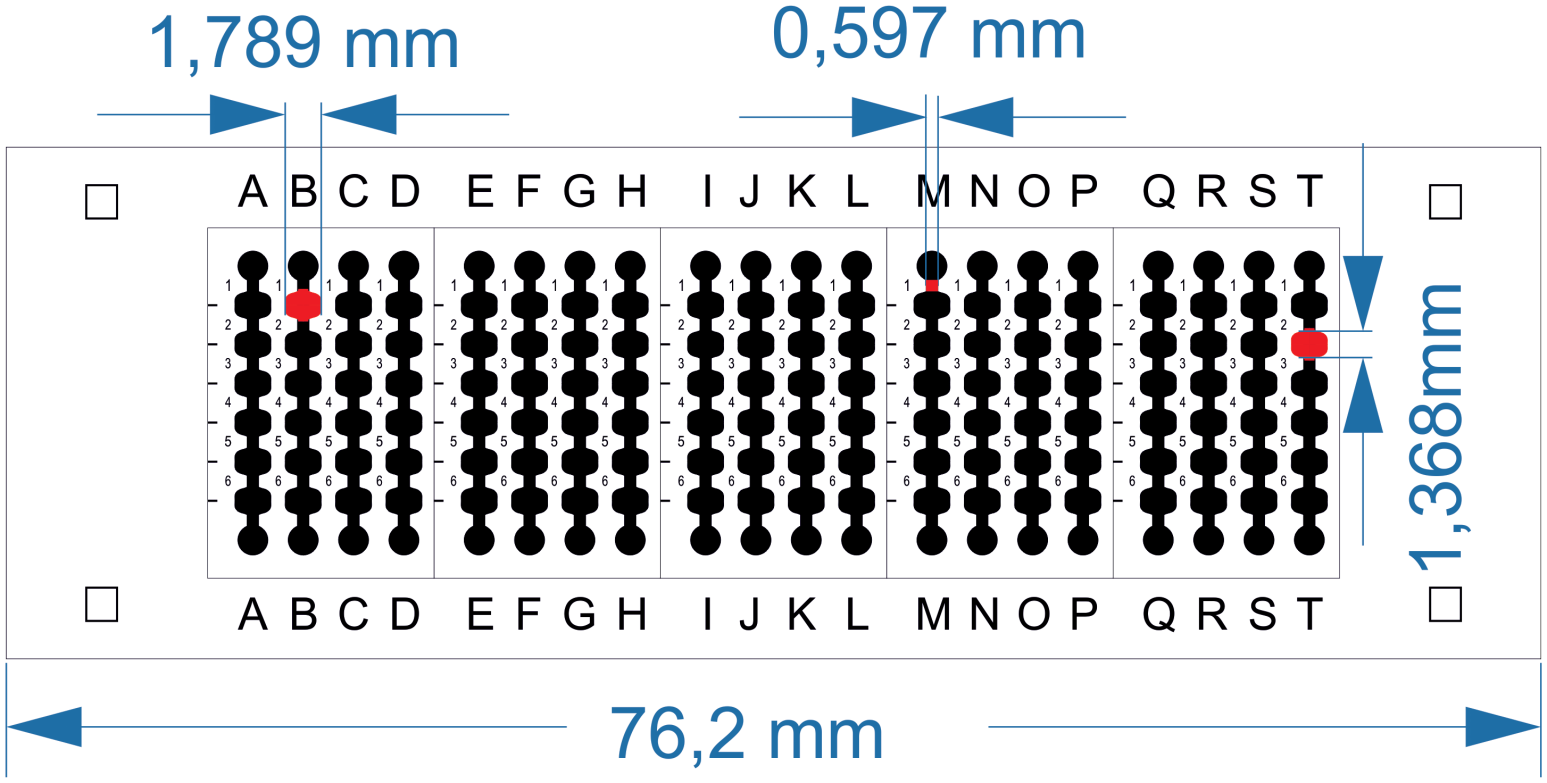
Microfluidic chip design. With 20 multichannels with 6 chambers per channel. The internal volume of each chamber is 130 nL.

A microfluidic device has been designed using Layout editor software (http://www.layouteditor.net). The microfluidic chip consists in 20 microchannels, with 6 chambers of 1, 368 mm wide and 13, 6 mm height. The internal volume is 130 nL (Fig 1).

The microdevices were built in PDMS. For this purpose, a mold of the design in high relief was made by photolithography in a silicon wafer 700 μm thick (Virginia Semiconductor, Inc.), by using the negative resin SU-8 (MicroChem). The silicon substrate was cleaned by sonication in acetone and isopropylic alcohol, and substrate surface was dehydrated for 10 min at 200°C. Then, SU-8 resist was dispensed on the substrate and spun in two cycles. The spinner was accelerated for 5 s at 100 rpm·s−1 until 500 rpm, and held at 500 rpm for 5 s. In the spin cycle, a ramp of 300 rpm·s−1 was applied until 2000 rpm, and held for 30 s. The resist was soft baked firstly at 65°C for 20 min, and secondly at 95°C for 50 min. The substrate was aligned and the resist was exposed to near UV at 650 mJ. The first step of a post-exposure bake consisted in 65°C for 12 min, and the second step at 95°C for 15 min. Finally, the resist was developed for 15 min under agitation.

**Fig 2:**
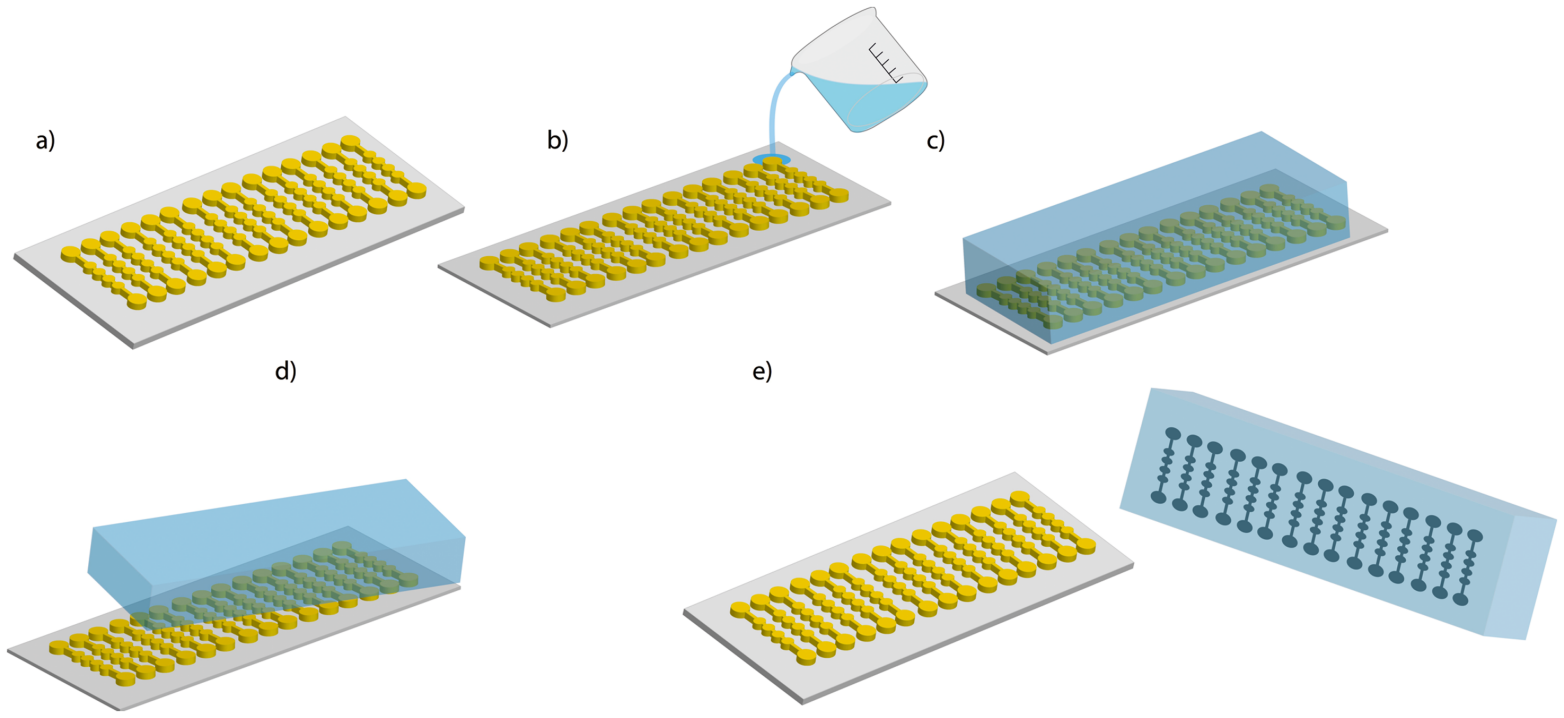
Microfluidic device synthesis. a) SU-8 resin on silicon wafer made by photolithography, b) PDMS poured over the mold, c) mixture of PDMS and curing agent (10: 1 ratio, 70 °C, 70 min), d) detachment of the mold.

PDMS (Sylgard 184, Dow Corning) was mixed with curing agent in a 10:1 ratio. The mixture was placed under vacuum to remove air bubbles. After this, the mixture was poured onto the master, placed under vacuum once again, and cured in an oven at 70°C for 70 min. Finally the devices were cut and the fluidic connection ports were punched with a syringe needle (21 gauge, 0,81 mm inner diameter). The device was then irreversibly bonded to a glass wafer after exposure to a high frequency generator (BD-10AS, Chicago, USA) for 120 s (Fig 2) [29].

### Cell culture and experimental design

The microfluidic chips were disinfected using NaOH 0.1mol·L^-1^ for 1 h, and then rinsed with sterile water. Before cell seeding, chip was treated with poly-D-lysine hydrobromide 0.1 mg·mL^-1^ (Sigma) sterile solution to improve cells attachment. The microdevice was incubated with poly-D-lysine solution for one hour at 37°C. The solution was then removed and let dry 24 h at 4°C. HEK-293T cells (ATCC CRL-3216) were cultured in complete DMEM medium (Gibco), supplemented with fetal bovine serum heat-inactivated (FBS) 10% (w/v) (Internegocios SA), L-glutamine 2mmol·L^1^ (Gibco), penicillin 100 units·mL^-1^, streptomycin 100 μg·mL^-1^ and fungizone 0.250 μg·mL^-1^ (Gibco) at 37°C in an incubator with 5% CO_2_. Cells were resuspended with trypsin 0.50 mg·mL^-1^ and EDTA-Na 0.2 mg·mL^-1^ (Gibco), and incubated at 37°C for 3 min. Trypsin was inactivated with FBS and cells were washed with phosphate buffer solution (PBS) (NaH_2_PO_4_ 50 mmol·L^-1^, NaCl 300 mmol·L^-1^, pH = 7.6) and centrifuged at 1000 rpm for 5 min. Finally, cells were resuspended in the same complete DMEM medium, supplemented this time with increasing concentrations of FBS (0; 1,25; 2,5; 5; 10 and 20%) at 500*10^3^ cells·mL^-1^ [30]. Each experimental condition presents 3 channels as triplicates with 8 chambers by each channel.

The microchannels were filled with 1 mL complete DMEM medium, and the system was purged for 5 min. Next, HEK-293T cells in suspension were seeded into the syringe needle in the inlet of the microfluidic device. Cells were allowed to settle and microfluidic device was incubated at 37°C in incubator overnight. A flow rate of 1 ml was applied with syringe to change DMEM medium every 48 h. The microchannels were visualized using an inverted Olympus microscope CKX41. Brightfield images were taken with Olympus objectives LUCPlan FLN 40×/0.60; LCAch N 20×/0.40; PlanC N 10×/0.25; and PlanC N 4×/ 0.10 with an Olympus QColor 5; and processed with QCapture Pro 6.0 software. Cell density inside microchannels was quantified first at the same time to seed the cells. After 24 h of incubation, cell density in each configuration was determined through quantification using the software for image analysis developed in this work, with images taken of 6× chambers acquired by triplicates. This analysis was done every 48 h approximately for 10 days. The results are shown as the mean ± standard error of the mean of data from 3 independent experiments, counting a total of 54 chambers for each concentration of FBS. The data were analyzed by single factor analysis of variance followed by the post hoc Dunn’s honestly significant difference test. A significance level of P<0, 05 was chosen, and SigmaPlot 12.5 (Systat Software, Inc) software was used for all statistical tests and line scatter analysis. The total number of images analyzed was 1000.

## Results and discussion

### Software data analysis

PIACG has been used to analyze the effects of increasing concentrations of FBS on HEK-293T cell line during 10 days of cell culture. It has allowed quantification of the area occupied by the cells in the well (%) in the microfluidic device in response to different concentrations of FBS, providing information on the rate of cell proliferation in response to FBS for 10 days of cell culture by triplicates by each experimental condition.

The first part of the image analysis is carried out by segmentation. Cell segmentation is the process by which each image is separated from the cell with respect to the background and other cells. The automated segmentation of cells is useful for the analysis of the cells obtained by both fluorescence microscopy and transmitted light, both in terms of objectivity and reduction of image analysis time for the researcher. This procedure allows automatic quantification of the characteristics of a large number of cells. Complete cell segmentation can provide information associated with individual cells. By segmentation, numerous cellular characteristics such as volume, shape or intercellular relations over time can be monitored. Automated analysis is a more scientifically objective method than manual image analysis and, therefore, improves reproducibility [31].

**Fig 3:**
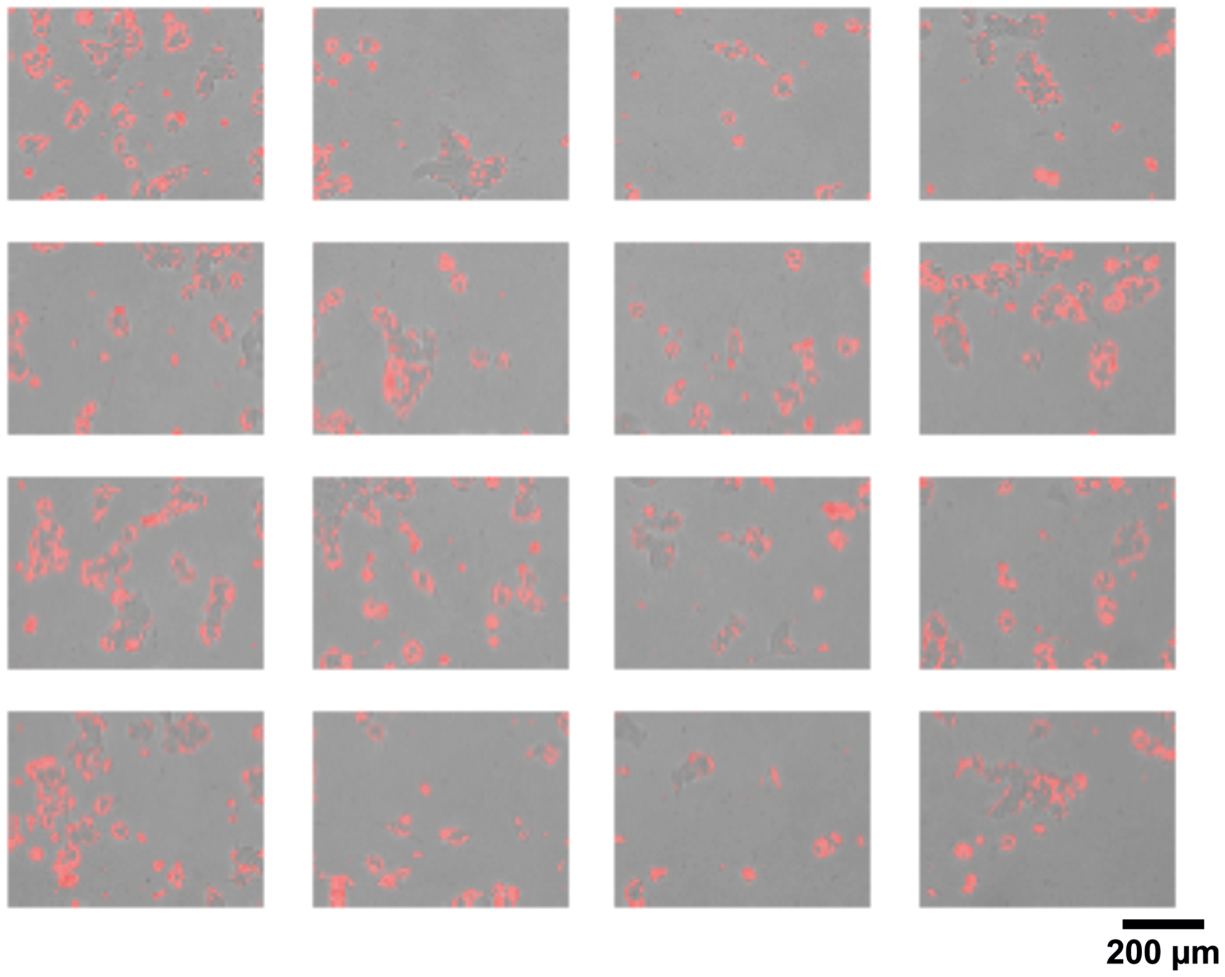
Segmentation of cells in a 2D cell culture. In red cell area detected by PIACG software respect to background. Images taken with transmitted light. Scale bar: 200 μm.

**Fig 4:**
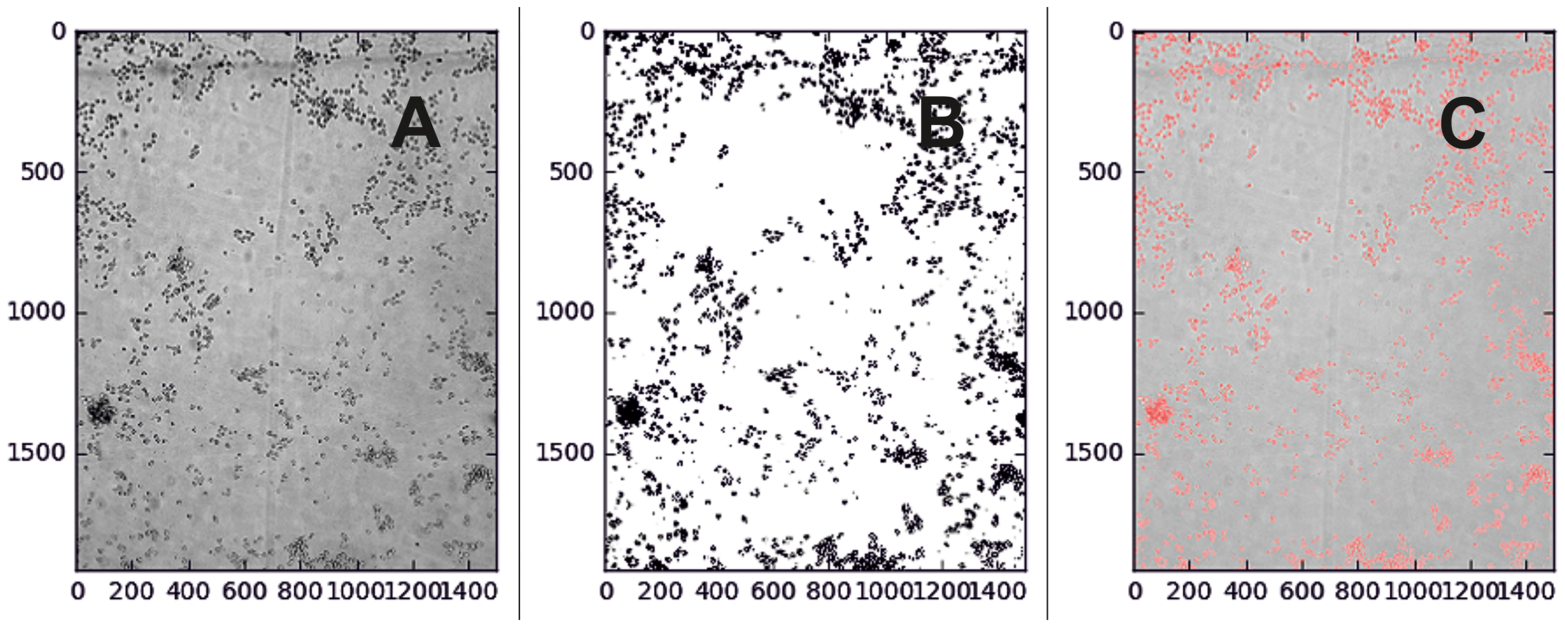
Segmentation procedure. A): Cellular area detected in gray scale. B): Threshold divides the whole image into two areas: cell / not cell (background). C): Cell occupied area is measured and compared to the original, safely *. * In this case, 11.05% of the image.

**Fig 5:**
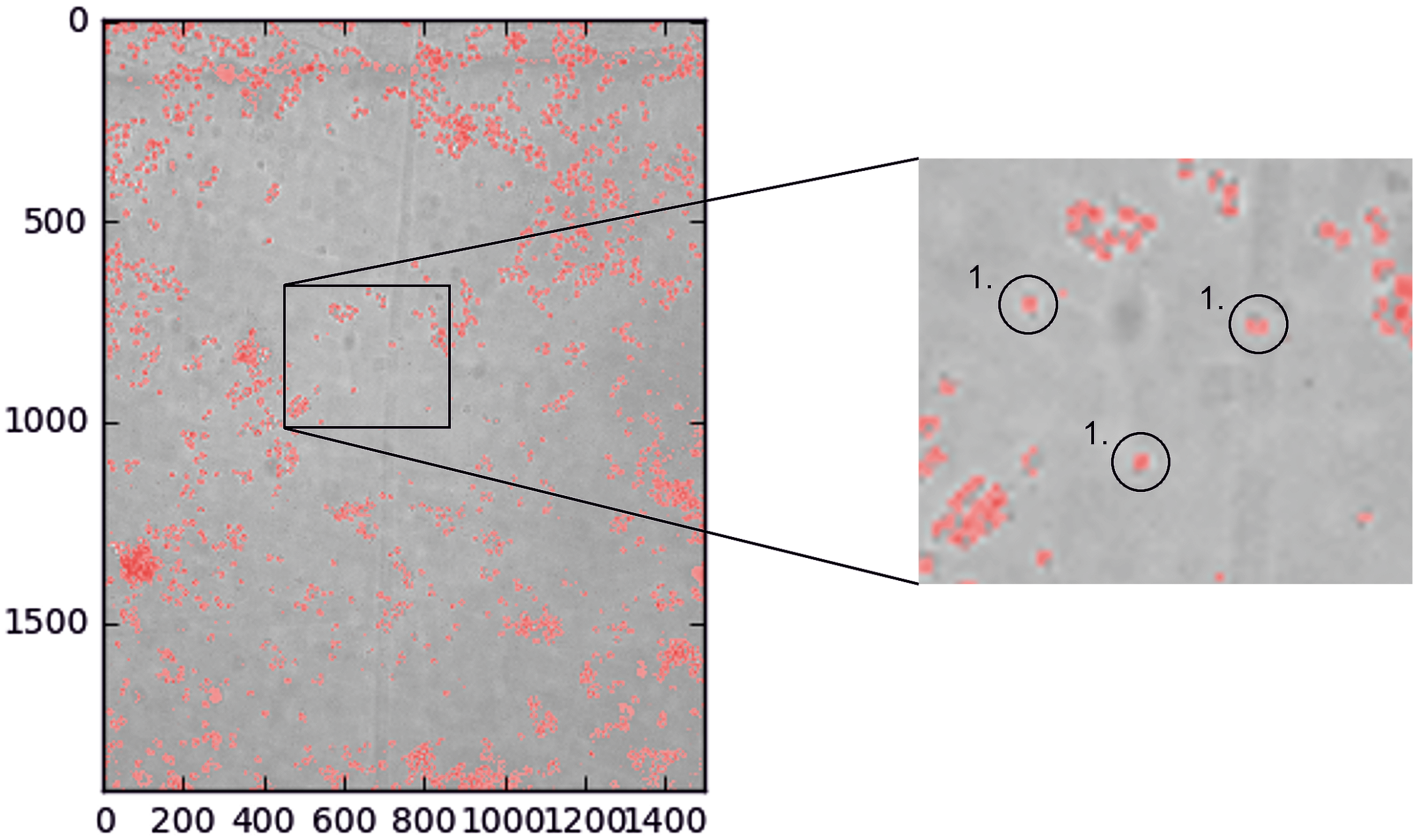
Determination of percentage cell area. From the determination of mean cell size, PIACG software estimates the mean height and cell area to calculate the percentage of total area occupied by the cells in the well.

Figs 3–5 present an example about how the system analyzes the obtained images, by cell segmentation, and calculates the area occupied by the cells in the chamber, estimating the number of cells existing in each experimental condition.

Segmentation cell applied to non-stained cells could minimize perturbation in the procedure due to the lack of chemical influence of a dye as well as the reduction of associated phototoxicity. However, some authors [31] argue that although segmentation in non-stained cells is extremely advantageous for individual cells, the boundaries are not readily captured, especially in cell cultures where the cells are densely clustered. To avoid these problems, the cell culture was maintained by controlling the population doubling to prevent the cell culture entering into confluence and difficult differentiation between specific cell boundaries.

Fig 4 shows how the software is able to discriminate the individual cells with respect to the background, converts to binary mode and marks the selected areas by comparing with respect to the background. This procedure allows calculating the single cell area and the total area of well occupied by the cell culture.

From single-cell segmentation, PIACG software first determines the average cell size. Subsequently, the estimation of the mean height and the total area of the cells are performed to determine the total area of well occupied by cells. Finally, software translates the dimensions into pixels in the metric system (Fig 5).

### Comparison between manual, ImageJ and PIACG software calculation

**Fig 6:**
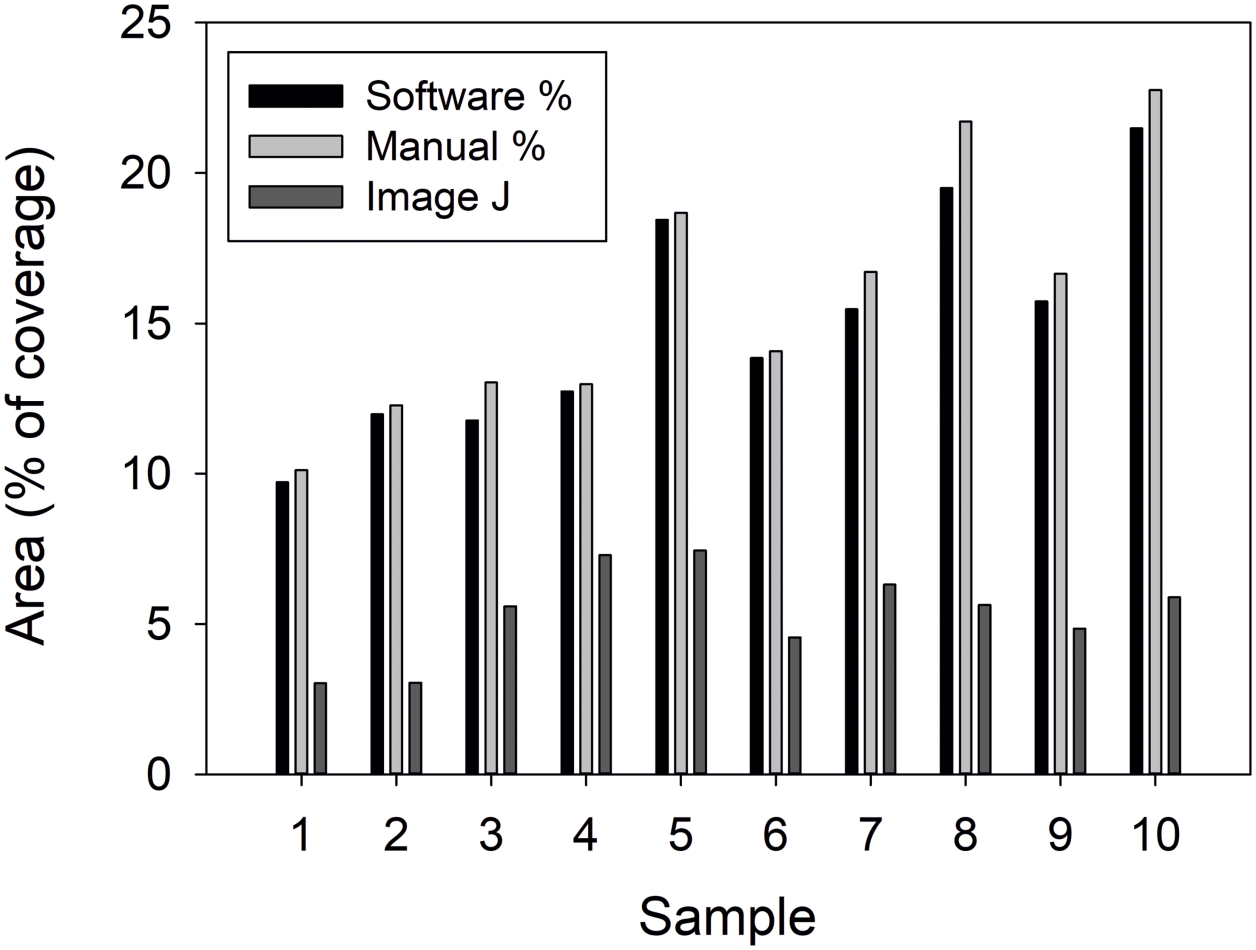
Comparison between manual, ImageJ and PIACG quantification. A bar graph comparing manual counts of cell area coverage versus to automated counts of the same images using PIACG and ImageJ. n = 10 images.

When it has been compared the cell area (%) obtained by manual counting with ImageJ and PIACG quantification (Fig 6), it can be seen that always, the areas quantified with the software are very similar to the areas quantified manually. It is due to the precise adjustment to the cellular morphology that the software achieves, reaching high accuracy in counting. However, counting with ImageJ shows that the quantized areas are always smaller compared to the areas quantified with PIACG software and manually. This is due to the need for pre-processing of images (brightness-contrast adjustments) required by ImageJ versus software quantification.

**Fig 7:**
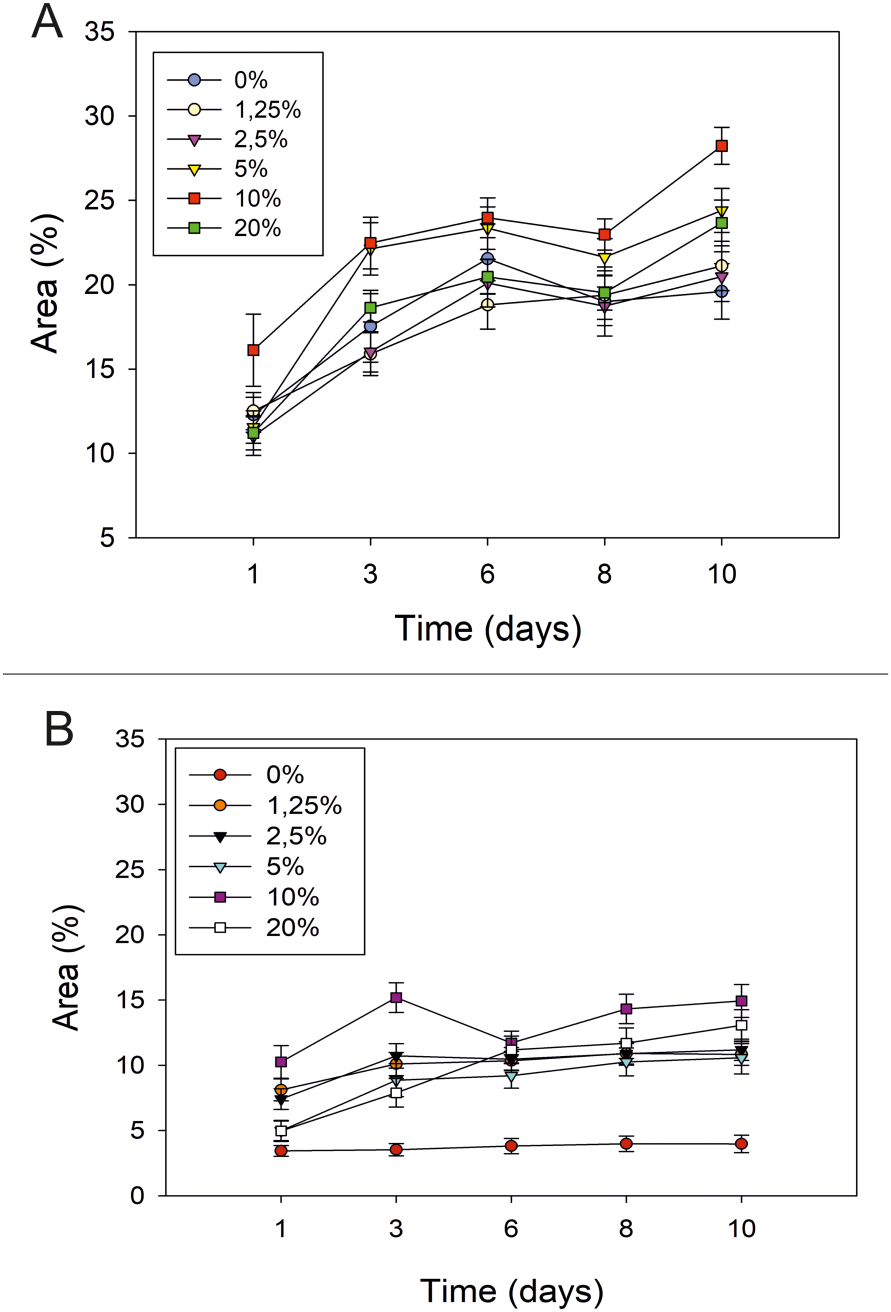
Comparison of cell detection accuracy with cell growth curves of HEK-293T cell line evaluated by PIACG and by ImageJ software. Comparison of cell detection accuracy evaluated by PIACG (A) and ImageJ software (B) analysis of cell growth curves of HEK-293T cell line treated with increasing concentration of FBS. HEK cells were inoculated into microfluidic device with growth medium containing 1,25: 2,5: 5; 10 and 20% FBS. The next day the cultures were washed with new cell culture medium. Images for cell area calculation were taken approximately every 48 hours, coinciding with the change of culture medium, until 10 days of cell culture.

It has been performed a comparative analysis with PIACG and with ImageJ software of the area occupied by cells in well in response to different concentrations of FBS during 10 days of culture (Fig 7). It has been compared the cell area accuracy detected by both software, using cell growth curves of cell line as comparative parameter.

As it can be seen in the figure, PIACG software presents a similar ability with respect to ImageJ software in the detection of the cellular area occupied in well in response to treatments with increasing concentrations of FBS. Cell growth curves have similar profiles in both analyzes.

From the biological point of view, the area has been used as a measure of the rate of cell proliferation. Cells subjected to treatments with 10% FBS during 10 days of cell culture are those that occupy the greater area of the well and therefore present higher rate of cell proliferation present. This result matches with the fact that 10% of FBS is used to supplement the culture medium for HEK cells in another type of substrates [30].

With the absence of FBS or with the lower concentrations treatment, a decrease in the area occupied by the cell culture in the chamber compared to 10% FBS concentration is observed and therefore, a reduction in the rate of cellular proliferation. This result is also detected in cells cultured with the highest concentration (20%) of FBS during 10 days of cell culture.

The results obtained by PIACG analysis match with the manual analysis of the images carried out (Fig 6), validating the effectiveness of the system.

To test the effectiveness of PIACG software, it was compared with ImageJ software. ImageJ [10] and Fiji [14] are very useful tools for image analysis and cell quantification.

PIACG software saves the pre-treatment of the image, saving time for the researcher. There are other image analysis software available such as BioImageXD [12] which is a tool oriented to the visualization in 3D of the cellular structures, allowing to perform slice, generation of structures in 3D, etc. Besides, Icy software [13] is a very complete tool. It allows the detection of cells and cell nuclei as spots, cellular segmentation, delimitation of the cell contour for cell tracking among other tasks in contrast phase images. Although, it requires a pre-processing step of the image or image batch in contrast phase images, where the quantification of spots is desired.

CellProfiler Analyst 2.0 [16] is able to perform a segmentation of the cells similar to PIACG software. Furthermore, it allows a classification by cell plotting. There is individual segmentation of cells, color and it classifies them separately. CellProfiler has filters to remove the images that do not accomplish the conditions, to put the type of treatment (experiment) as PIACG software.

In addition, Vaa3D [15] is a fast, and versatile 3D/4D/5D Image Visualization and Analysis System for Bioimages. Whereas, Advanced Cell Classifier [32] sorts cell phenotypes by immunofluorescence. Indeed, it executes a type of flow cytometry through microscopy and immunofluorescence since it classifies the cells by size vs. size or size vs. complexity. So far, the PIACG software has been proposed as a tool for the quantification of the area occupied in cell cultures of a specific cell type. A plugging could be implemented in the future to discriminate between cellular phenotypes in mixed cell cultures by segmentation.

As mentioned previously in the Introduction section, there are several analytical tools already on the market. However, many of them, despite being useful for the processing of fluorescence and phase contrast images, often do not provide good results from transmission light microscopy images, due to the intrinsic variation of the acquisition technique itself and the variability introduced between image acquisition by operators and by equipment [25]. A new method to restore artifact-free microscopy images that are suitable for segmentation by direct thresholding is presented [33]. It is oriented to count the total number of cells, so it does not make a complete segmentation of the cell morphology, only of the soma, not delineating the complete structure of the cell. Although it generates a comparison in phase contrast images, the PIACG software is able to segment cell morphology completely, quantifying another parameter of interest: the total area occupied by cell population in the well and therefore the cell confluence in a cell culture device.

With respect to the cell growth curves obtained in this work in microfluidic chips compared to another kind of substrates, some studies [34] have shown that the rate of cell proliferation varies significantly between cultures in microfluidic chips and macroculture. These authors observe a significant inhibition of the rate of cell proliferation in the microfluidic devices compared to the macrocultures and explain this fact due to the increase in the metabolic rate, increment of cellular stress markers as well as cell / media interactions with the material of the microfluidic device that they observe in the microfluidic chip cell cultures against the cell cultures in other type of supports.

### Biological analysis

**Fig 8:**
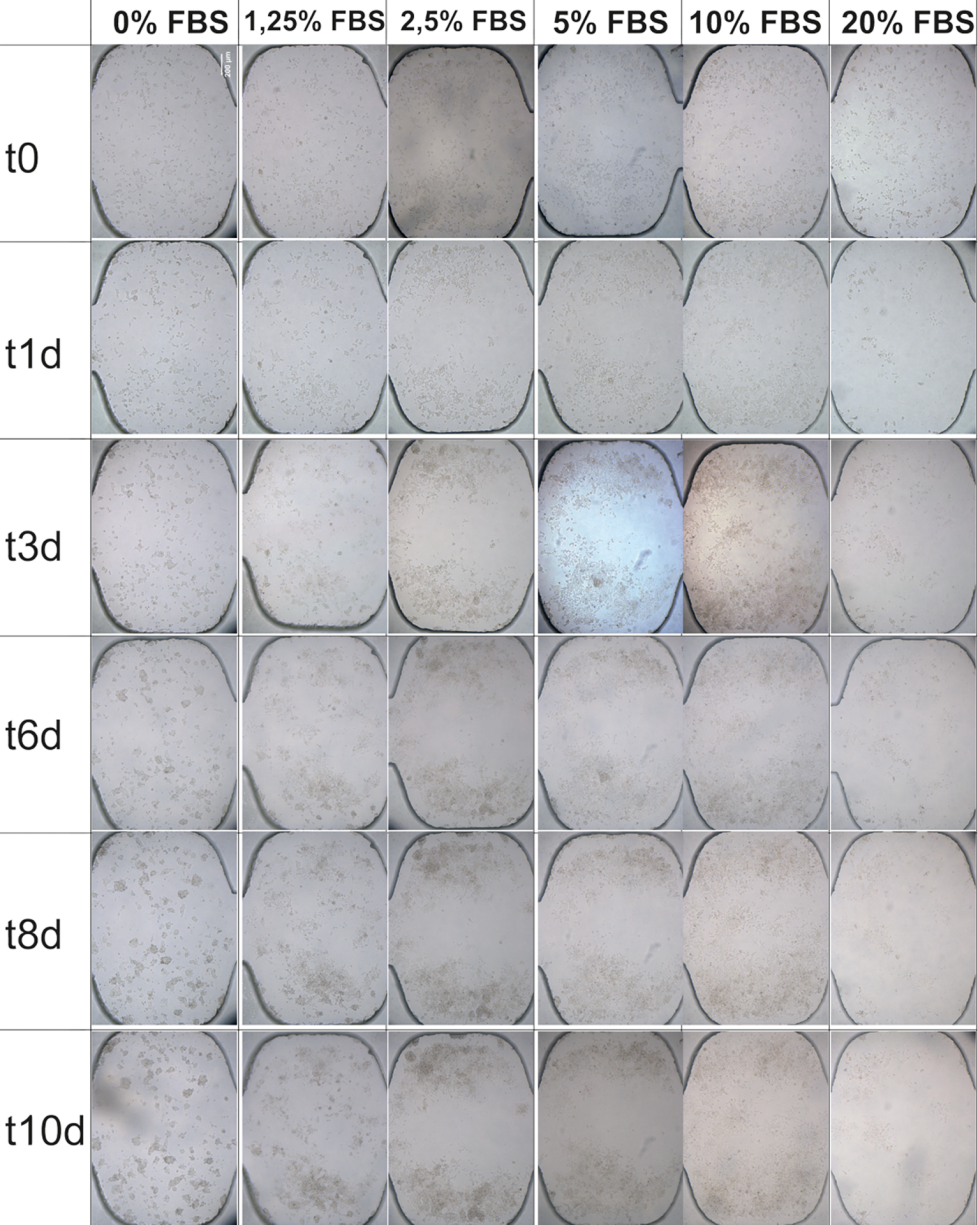
Comparative analysis of effects of FBS concentrations on HEK-293T cell line. Phase contrast images of HEK cells during 10 days of cell culture in microchannel across a range of serum concentrations. Brightfield images were taken by inverted Olympus microscope CKX41, with Olympus objective PlanC N 10×/0.25. Cells were seeded at a density of 500*10^3^ cells.ml^-1^ and cultured in DMEM cell culture medium. The images are representative of 6 chambers and 3 channels from 3 independent experiments. Scale bar: 200 μm.

**Fig 9:**
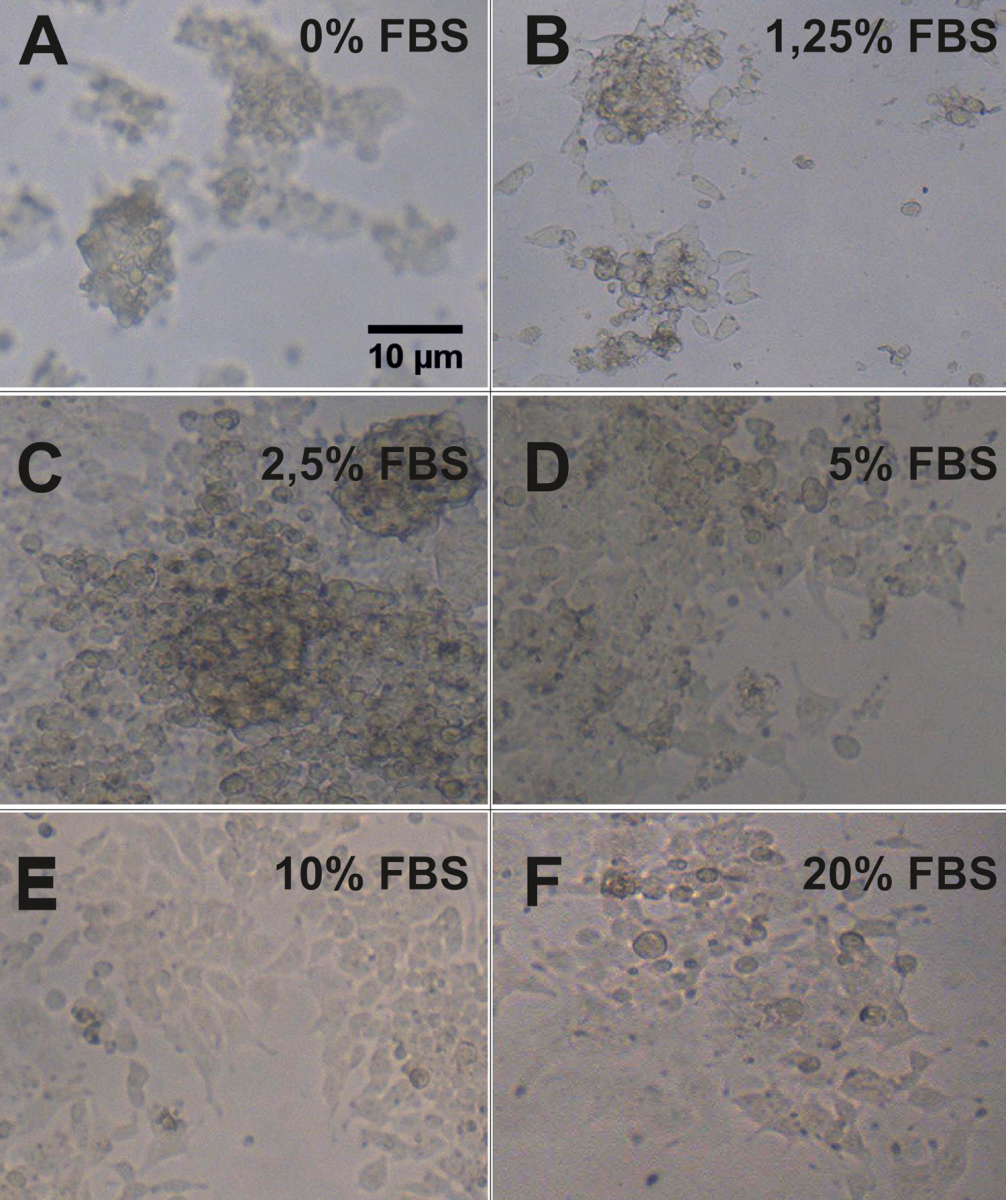
Effects on cell attachment of concentration of FBS in HEK-293T cell line. Magnification of phase contrast images of HEK cells with increasing concentrations of FBS. The figure shows the effects that long exposure with several concentrations of FBS can make on attachment of cells to the substrate, probably due to the effects it exerts on the anchor/cytoskeleton proteins of cells [35]. The images show an area of the same field that pictures showed at Fig 8, at a higher magnification. Scale bar: 10 μm.

A comparative analysis of the different percentage of FBS used for each day of culture (Fig 8), shows that the lack of serum (0% FBS) does not affect cells during the first until the third day of culture. However, it seems to affect from day number 6 of cultivation since cytoskeletal disorganization is observed, cells begin to aggregate and form clusters as a result of the loss of adhesion to the substrate (Fig 9A).

Treatments with serum percentages below 5% (1.25 and 2.5%) do not affect cell proliferation during the first day of culture. However at the third day, the formation of cell clusters is observed as a consequence of the loss of adhesion to the substrate by the disorganization of the cytoskeleton (Fig 9B and 9C).

When the culture medium contains 5% FBS, a normal cell proliferation rate is observed. The cells have an organized cytoskeleton and good cell adhesion. However, after 3 days of cell culture, a high cell density is observed simultaneously with signs of disorganization of the cytoskeleton that appear in numerous cells and consequently the formation of clusters due to the lack of adhesion to the substrate (Fig 9D). With 10% serum treatment, cells maintain optimal cell proliferation rate and cell density throughout the cell culture, maintaining adherence to the substrate and cell organization at all times (Fig 9E).

If the culture medium contains 20% FBS, it is observed that after 1 day of culture the adhesion of the cells to the substrate is lost, decreasing the cell density in the well. However, after 3 days of culture it is observed that the cells that remain attached to the substrate begin to divide with a normal rate of cell proliferation (Fig 9F).

All effects described for treatments with different serum concentrations have been observed in all experimental replicates that have been made.

## Conclusions

While manually quantification and other image analysis software require optimal conditions of brightness / contrast in the image, and therefore, the previous manipulation of the images to be quantified, PIACG allows the quantification of the cellular area without the need for previous adjustments in the images, saving the researcher a considerable time of analysis and offering more accurate results. Further, the software developed in this work allows an automatic analysis and cell quantification of hundreds of pictures simultaneously. It aims to offer a general solution, and considerable time saving in data analysis, through a simple and sensible user interface.

In future studies, the cells will be cultured for longer period of time to see if there is a significant difference in the treatment at 10% of FBS with respect to the other treatments. The software will also be optimized to calculate cell viability, differentiating living cells from dead cells by color and morphology. Statistical analysis of data/images obtained under different culture conditions will be performed employing a multichannel input system to adjust the various culture components that should allow optimizing cell culture parameters. Using AI techniques to analyze the data and to modify culture conditions accordingly, it should facilitate a more rapid development of optimum culture conditions for a variety of different cell types and application in production, in research, and in clinical application.

## Supporting information

S1 File PIACG software code. (ZIP)
