# Supplementary material for: Development of image analysis software for quantification of viable cells in microchips
Source: PLoS One. 2018 Mar 1;13(3):e0193605. doi: 10.1371/journal.pone.0193605 (PMC5832319; doi:10.1371/journal.pone.0193605)
Supplement: S1 File — (ZIP) [file pone.0193605.s001.zip › source_code/manual.rtf]

How to use the image analysis tool for cell growth experiments:


Arranging the input images:

1. Open the „cell growth“ folder
2. Open the input folder
3.1. Inside the input folder create a folder for every day of your experiment at which you took pictures
3.2. Label them just with number (e.g. 0 if you took pictures at the very beginning or 2 if you took pictures after 2 days)
4.1. Inside one of these folders create a folder for each different concentration you used
4.2. Label them just with numbers, use „ . “ not „ , “ if the have a fractional part, don't add „ % “ or anything else that is not a number
4.3. Copy those folders to all day-folders
5. Sort your images in the created folders, it doesn't matter how they are labeled (I recommend to use .jpg but it should work with any format).


Starting the analysis:

1. Navigate to the folder „image analysis tool“
2. Open the Terminal (press cmd+space, type terminal, press enter)
3. In the Terminal type „cd “ (without quotation marks, don't forget the space, don't press enter yet)
4. Drag and drop the „cell growth“ folder into the Terminal window
5. Press enter and follow the instructions (if you want to change some values open the user_input.py file, read the instructions an make your changes).


(The code file has been placed in https://github.com/maximiliangeorg/mf_img_analysis)
