# Supplementary figures and images for: Development of image analysis software for quantification of viable cells in microchips
Source: PLoS One. 2018 Mar 1;13(3):e0193605. doi: 10.1371/journal.pone.0193605 (PMC5832319; doi:10.1371/journal.pone.0193605)

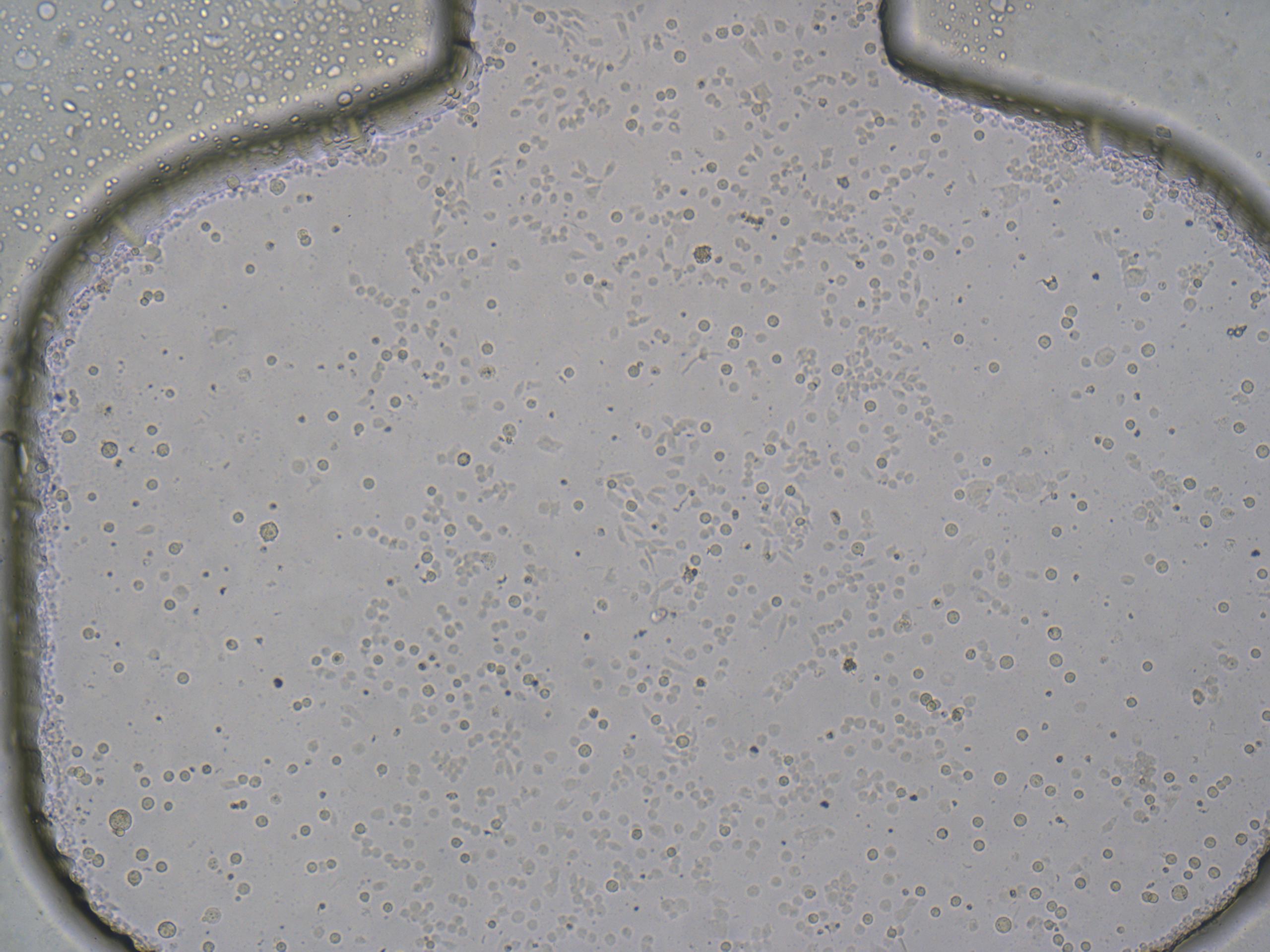

Supplement: S1 File — (ZIP) [file pone.0193605.s001.zip › source_code/input/0/1.25/2016-11-18-D1-8e5hek.jpg]

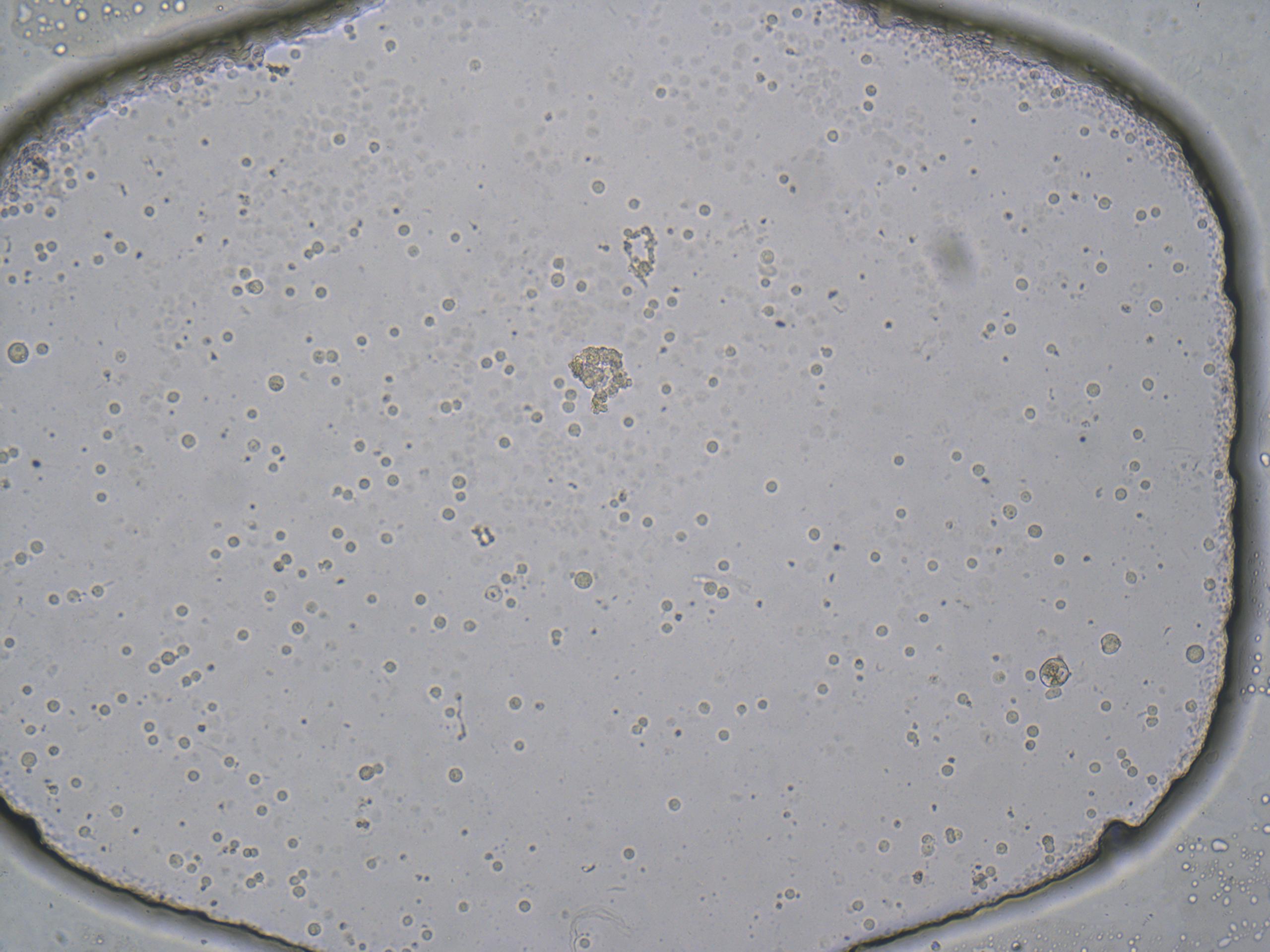

Supplement: S1 File — (ZIP) [file pone.0193605.s001.zip › source_code/input/0/1.25/2016-11-18-D2-8e5hek.jpg]

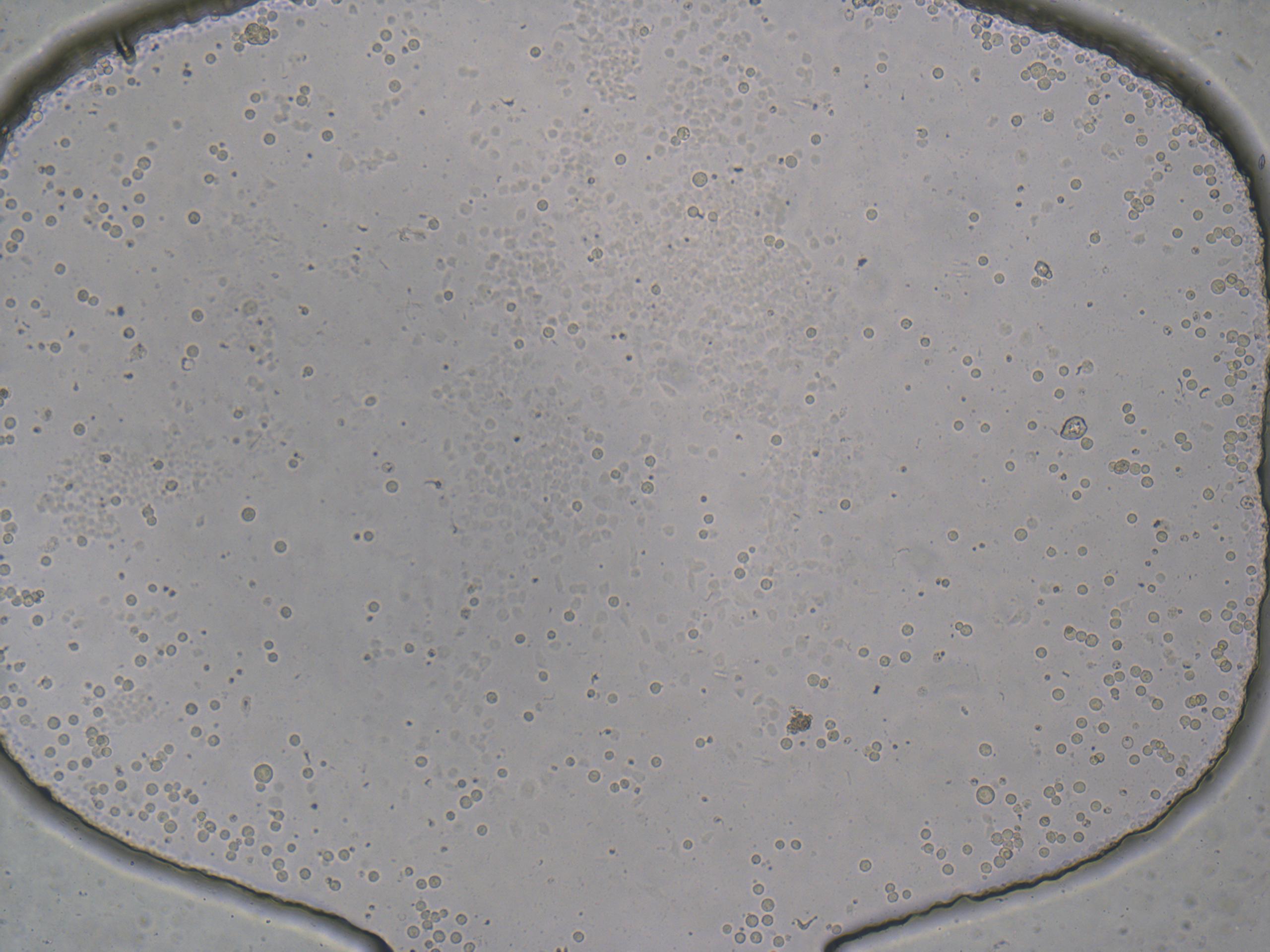

Supplement: S1 File — (ZIP) [file pone.0193605.s001.zip › source_code/input/0/2.5/2016-11-18-G1-8e5hek.jpg]

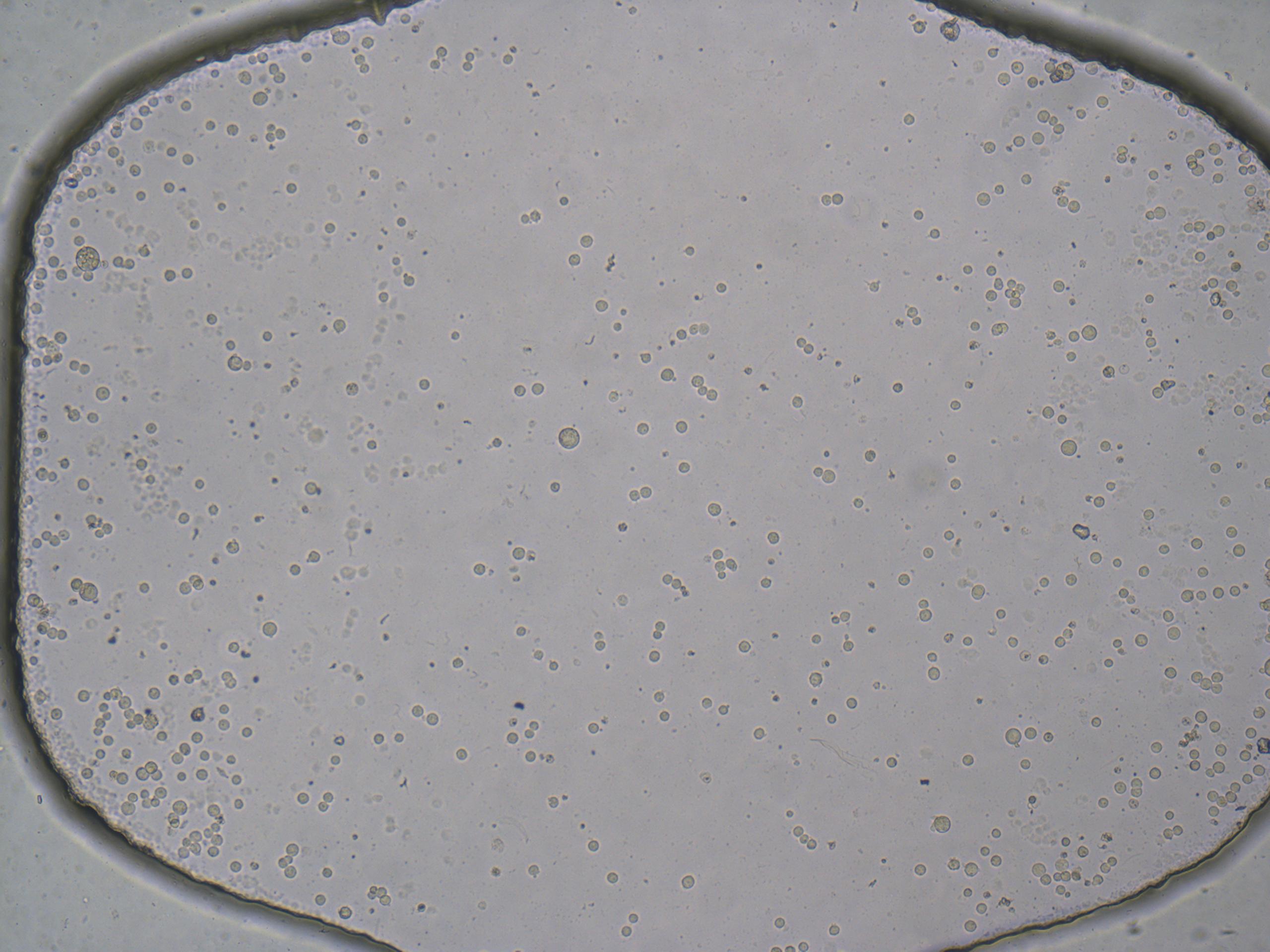

Supplement: S1 File — (ZIP) [file pone.0193605.s001.zip › source_code/input/0/2.5/2016-11-18-G2-8e5hek.jpg]

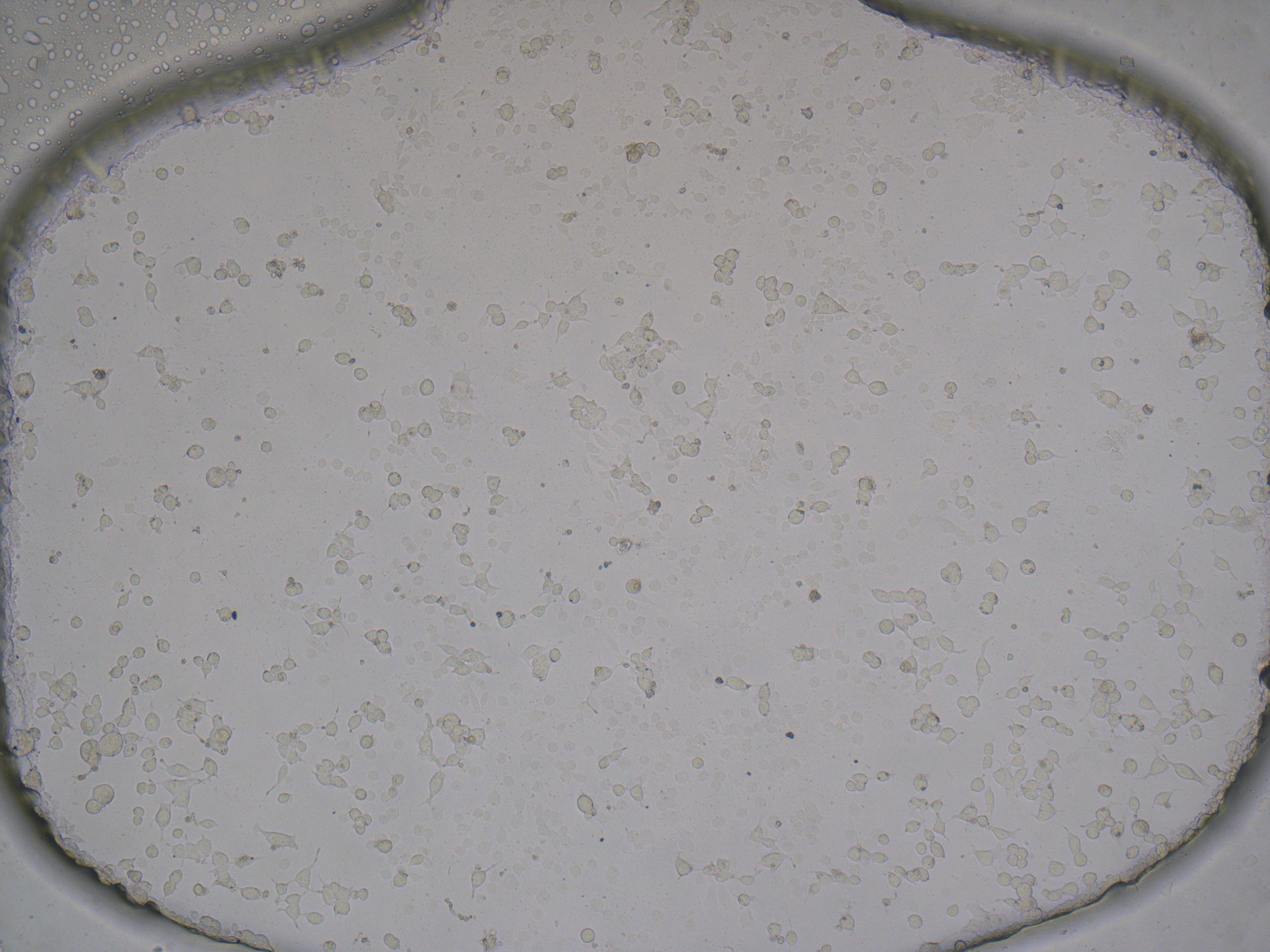

Supplement: S1 File — (ZIP) [file pone.0193605.s001.zip › source_code/input/3/1.25/2016-11-21-D1-8e5hek.jpg]

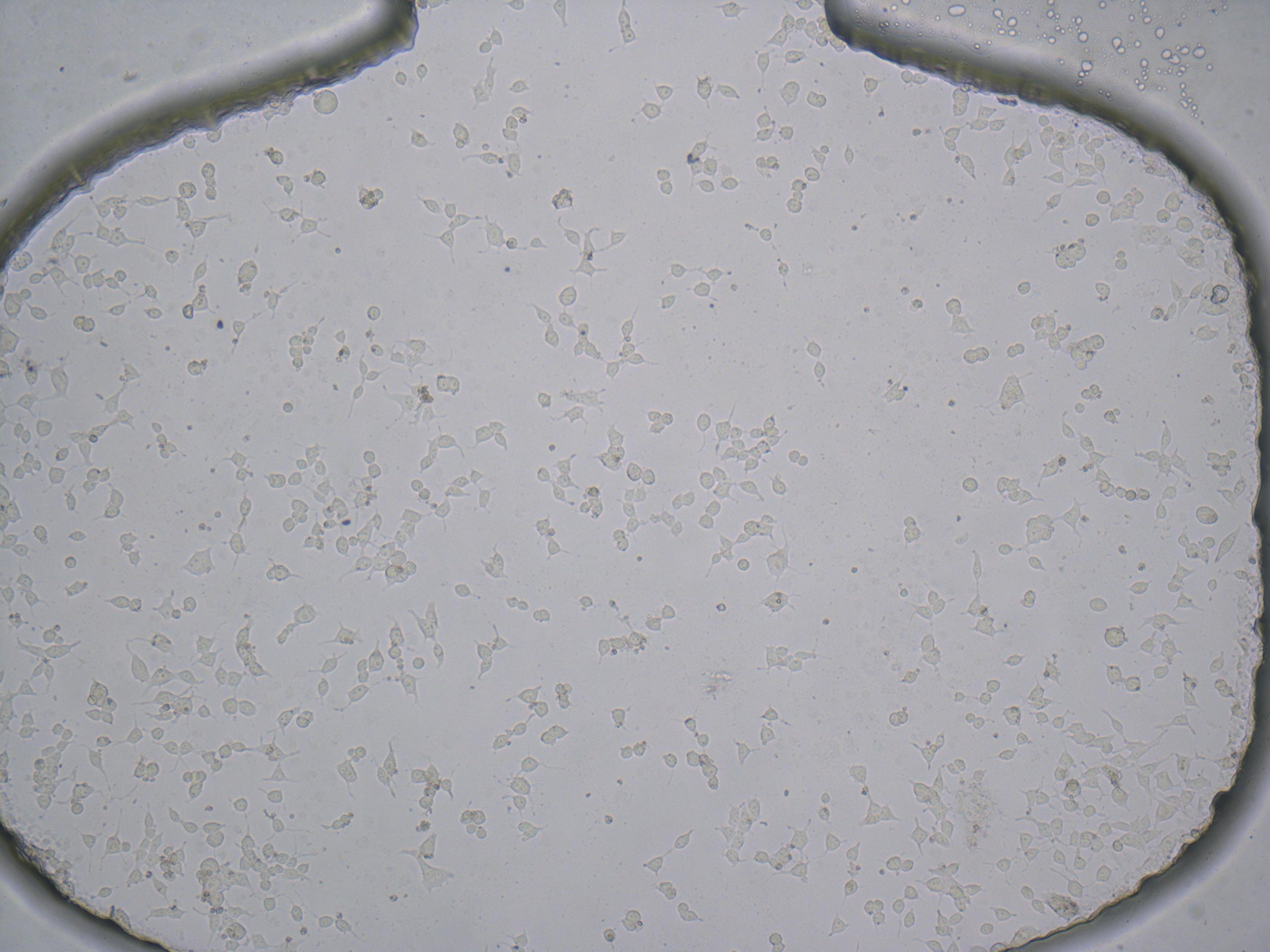

Supplement: S1 File — (ZIP) [file pone.0193605.s001.zip › source_code/input/3/1.25/2016-11-21-D3-8e5hek.jpg]

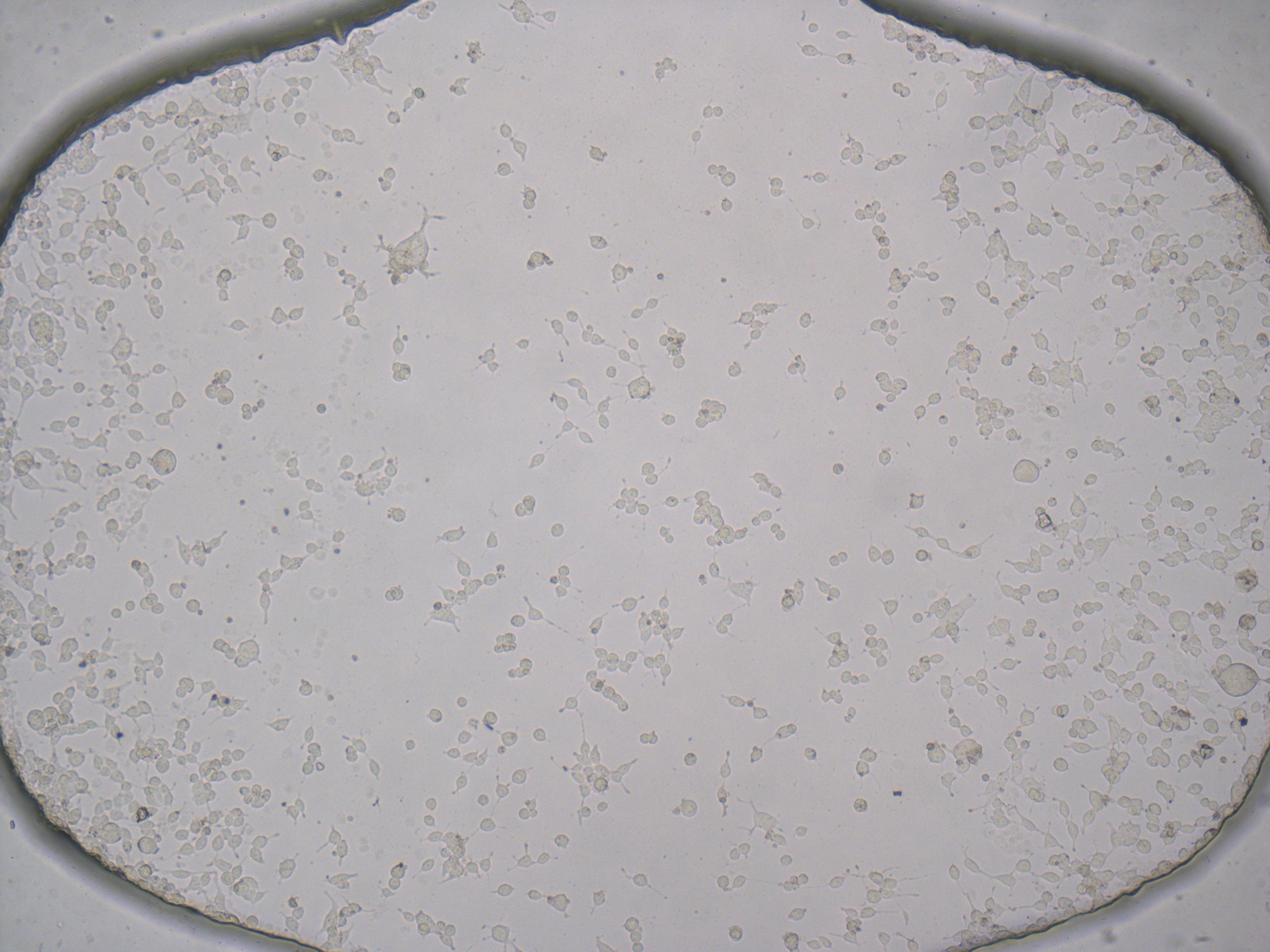

Supplement: S1 File — (ZIP) [file pone.0193605.s001.zip › source_code/input/3/2.5/2016-11-21-G2-8e5hek.jpg]

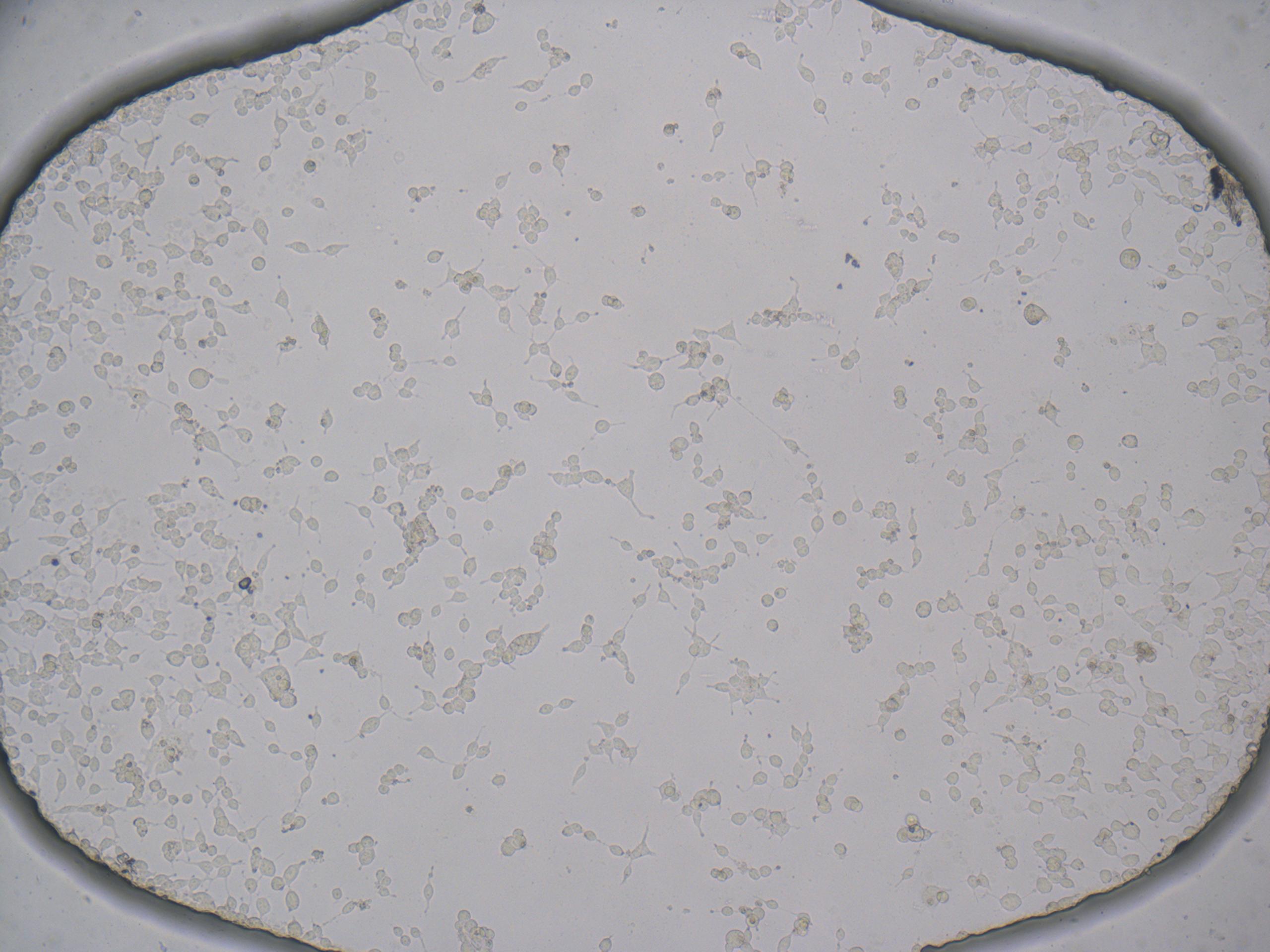

Supplement: S1 File — (ZIP) [file pone.0193605.s001.zip › source_code/input/3/2.5/2016-11-21-G4-8e5hek.jpg]

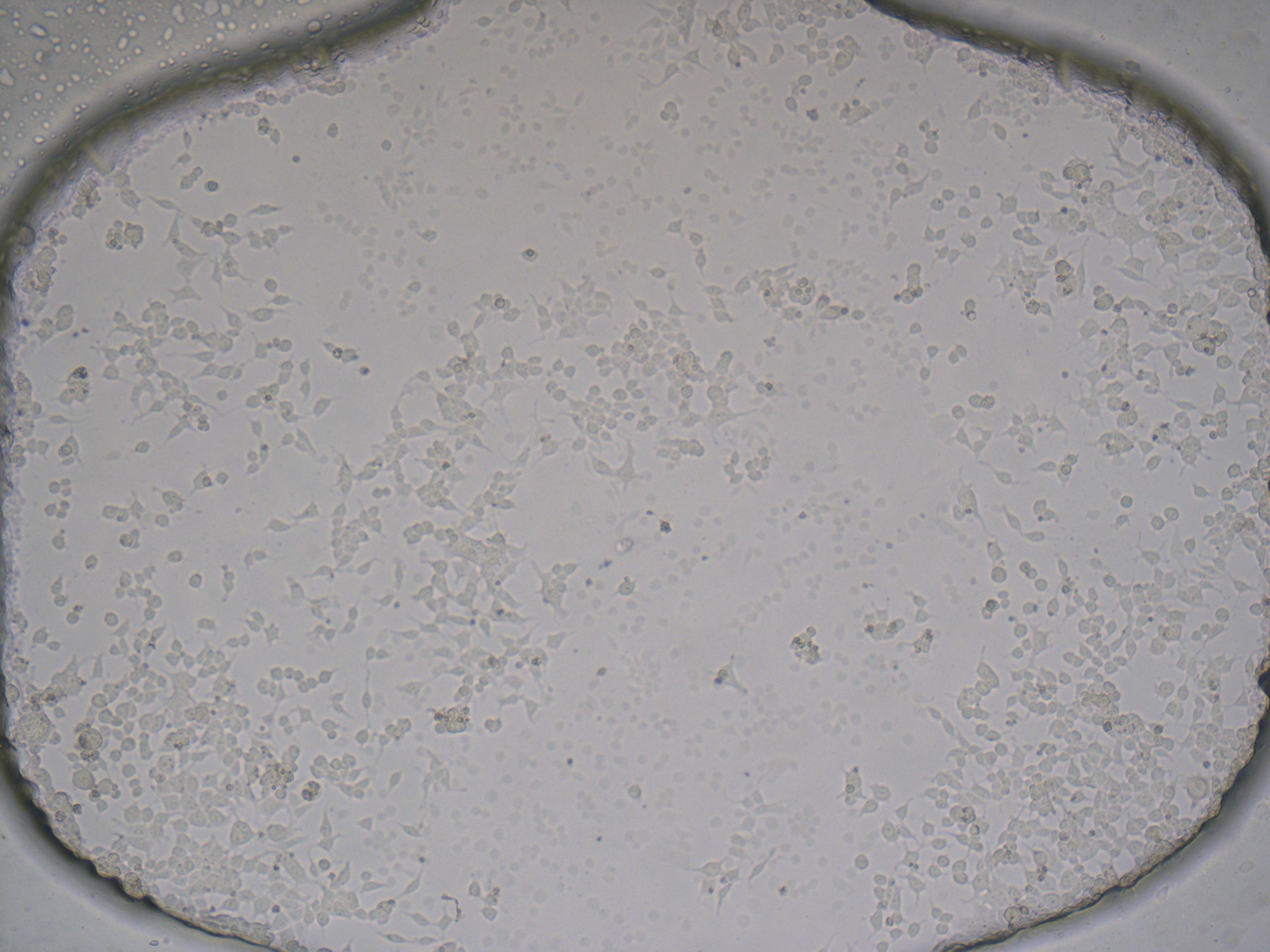

Supplement: S1 File — (ZIP) [file pone.0193605.s001.zip › source_code/input/5/1.25/2016-11-23-D1-8e5hek.jpg]

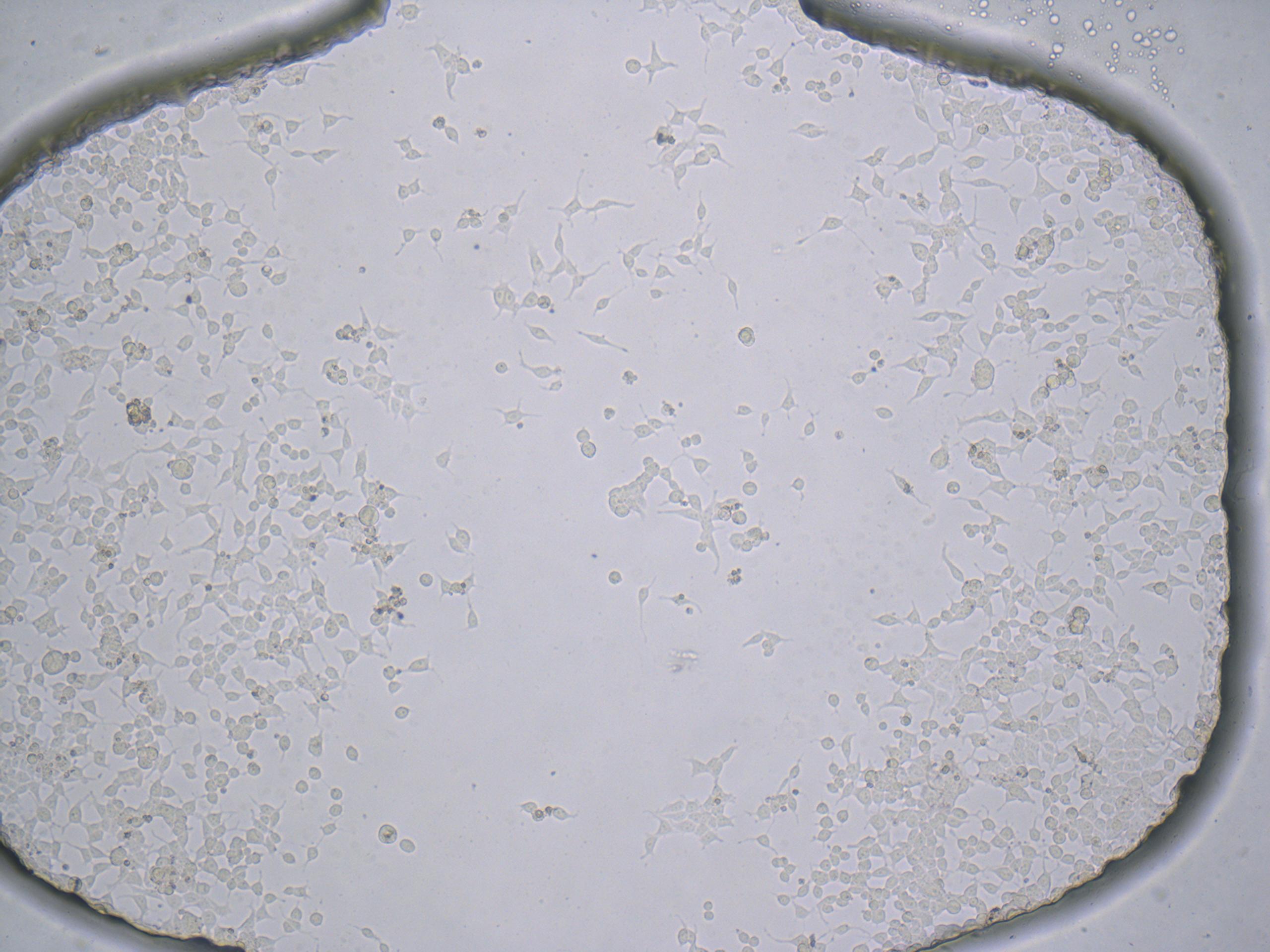

Supplement: S1 File — (ZIP) [file pone.0193605.s001.zip › source_code/input/5/1.25/2016-11-23-D3-8e5hek.jpg]

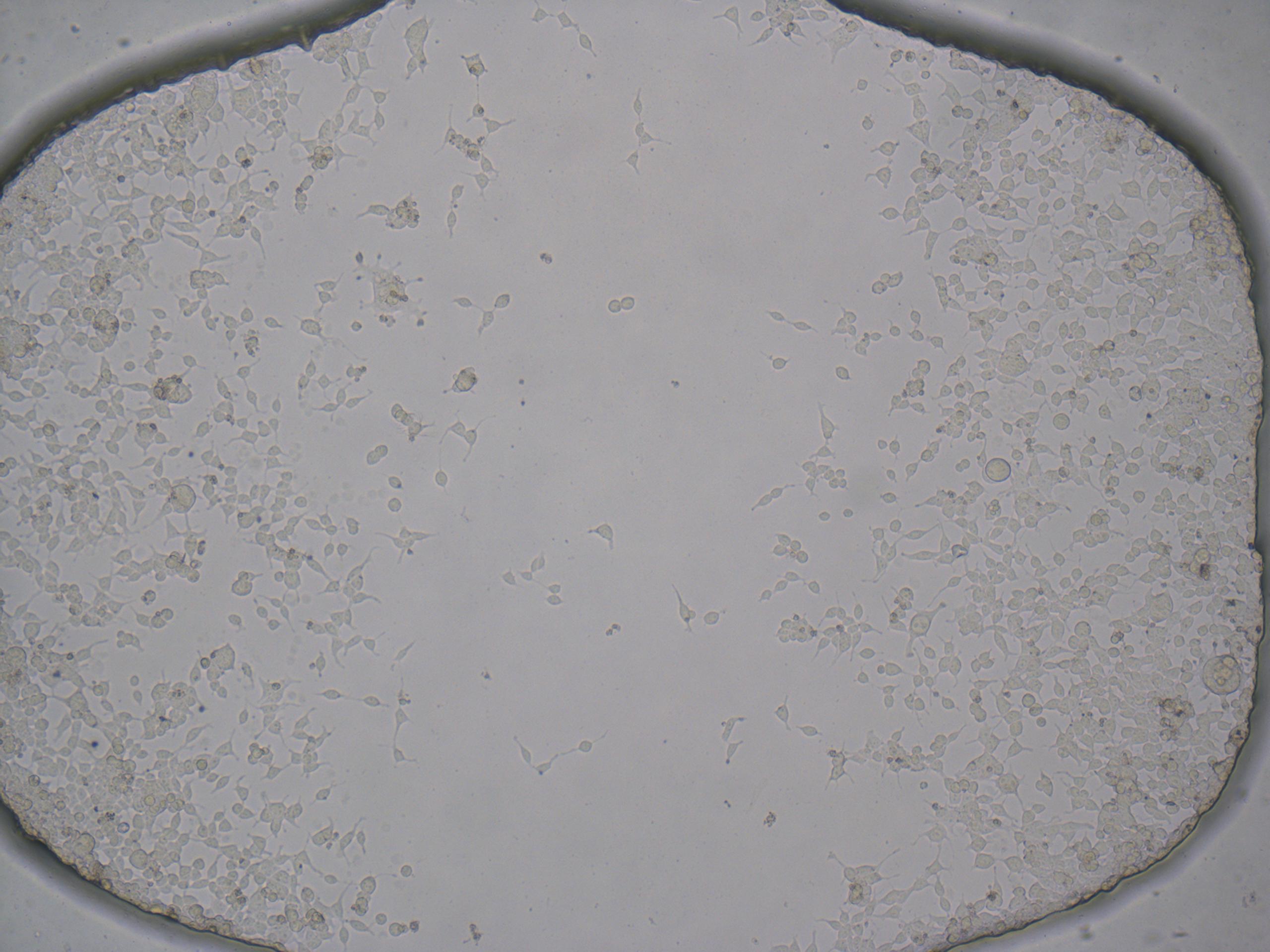

Supplement: S1 File — (ZIP) [file pone.0193605.s001.zip › source_code/input/5/2.5/2016-11-23-G2-8e5hek.jpg]

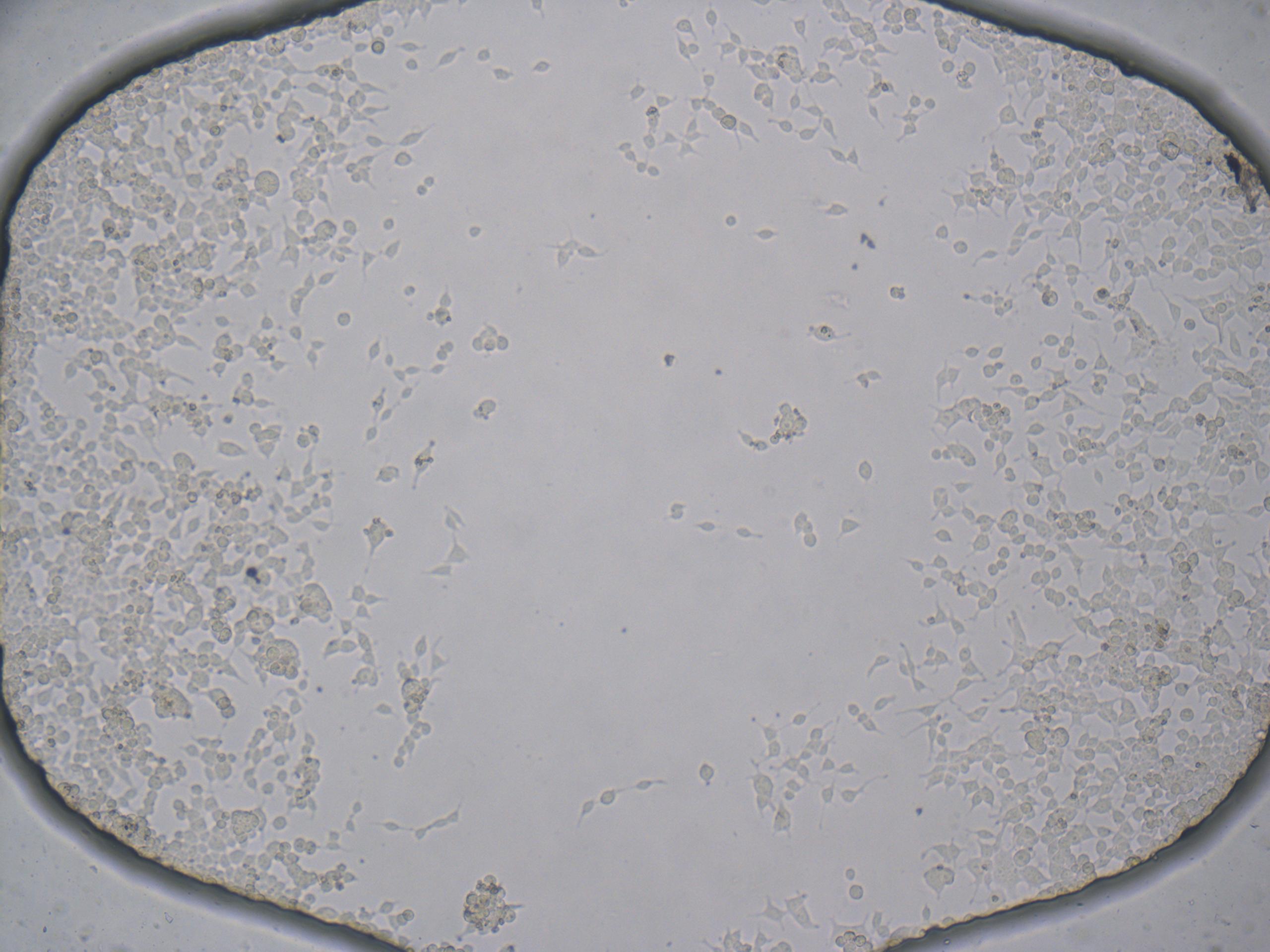

Supplement: S1 File — (ZIP) [file pone.0193605.s001.zip › source_code/input/5/2.5/2016-11-23-G4-8e5hek.jpg]

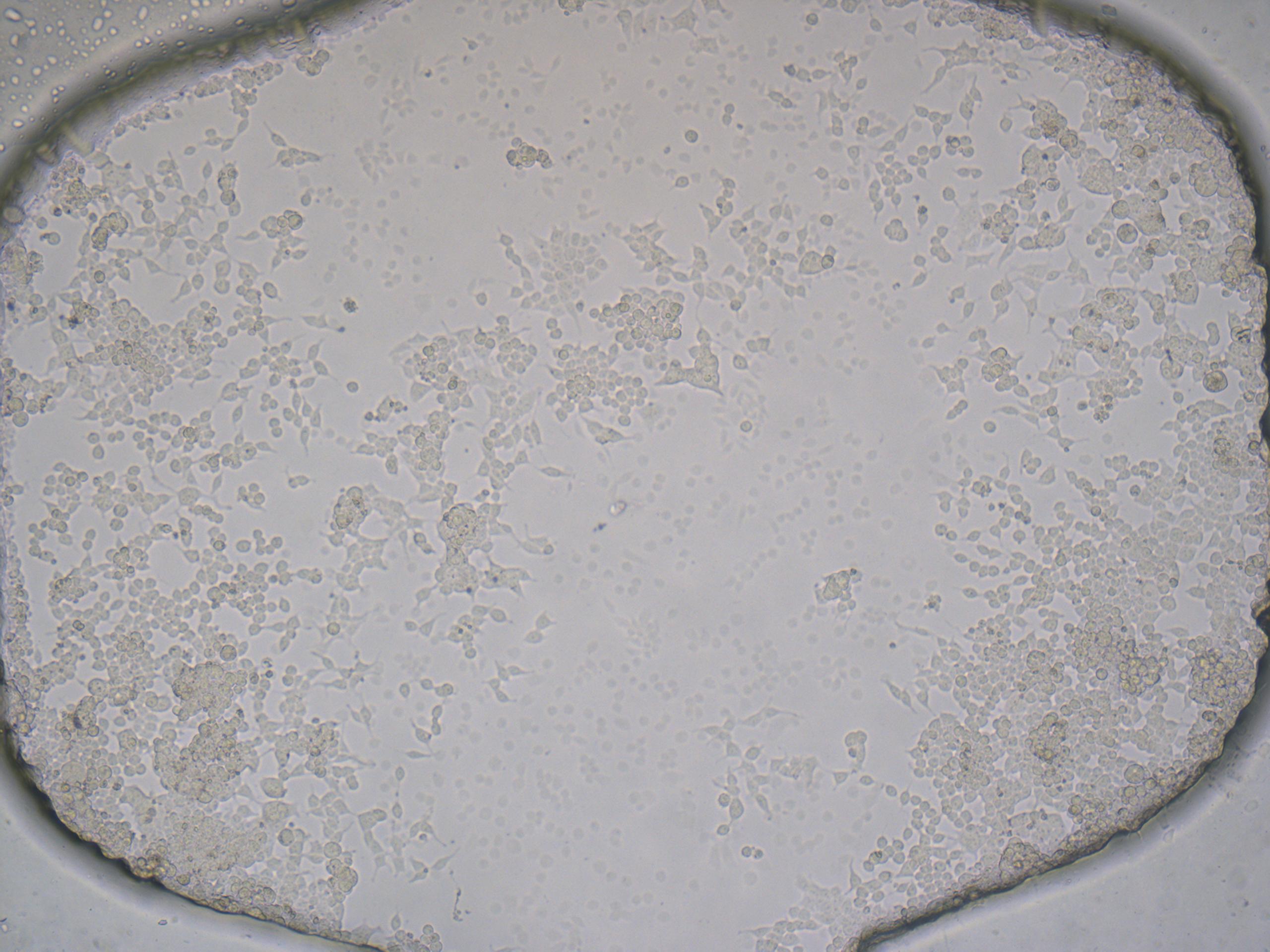

Supplement: S1 File — (ZIP) [file pone.0193605.s001.zip › source_code/input/7/1.25/2016-11-25-D1-8e5hek.jpg]

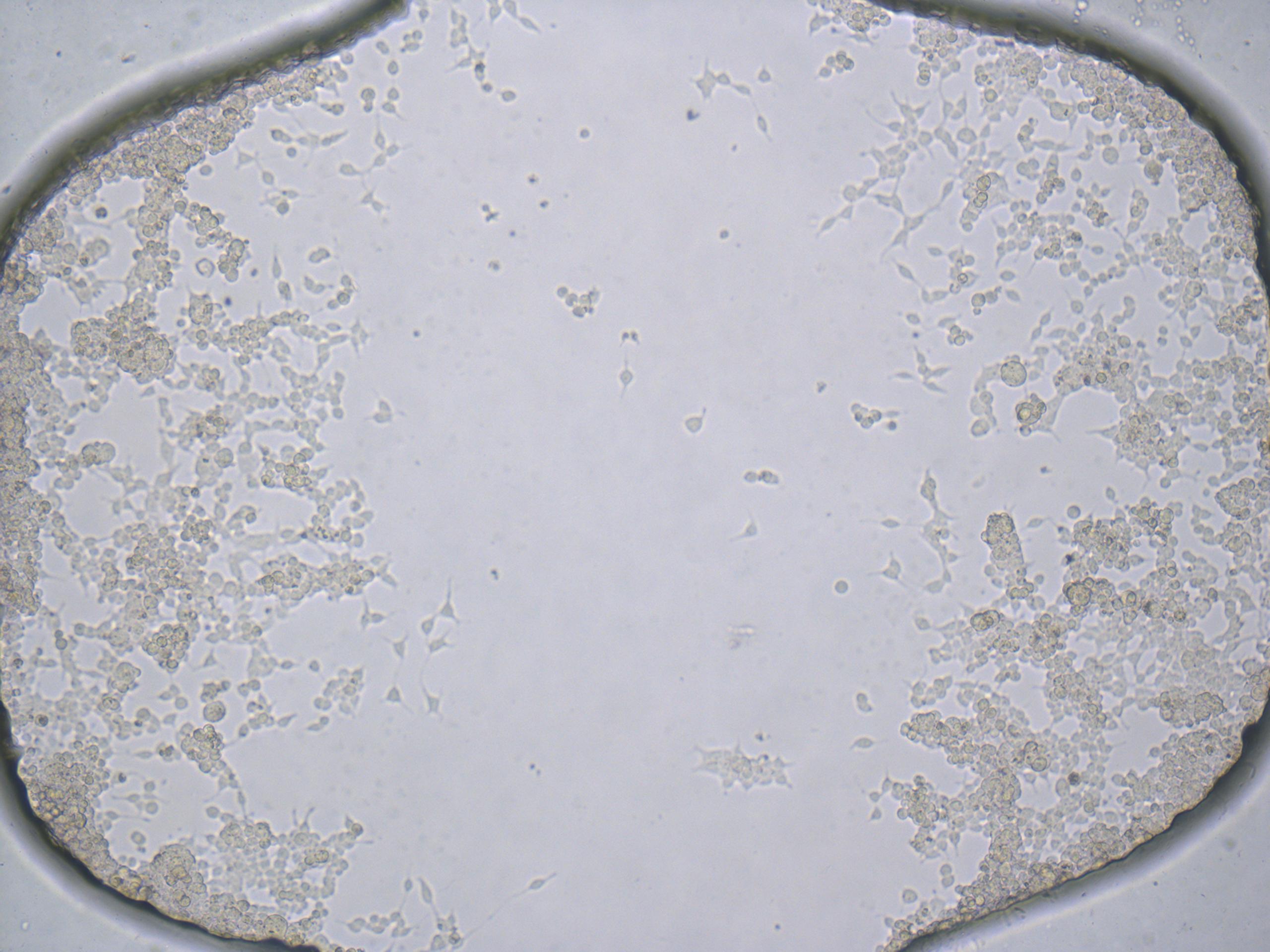

Supplement: S1 File — (ZIP) [file pone.0193605.s001.zip › source_code/input/7/1.25/2016-11-25-D3-8e5hek.jpg]

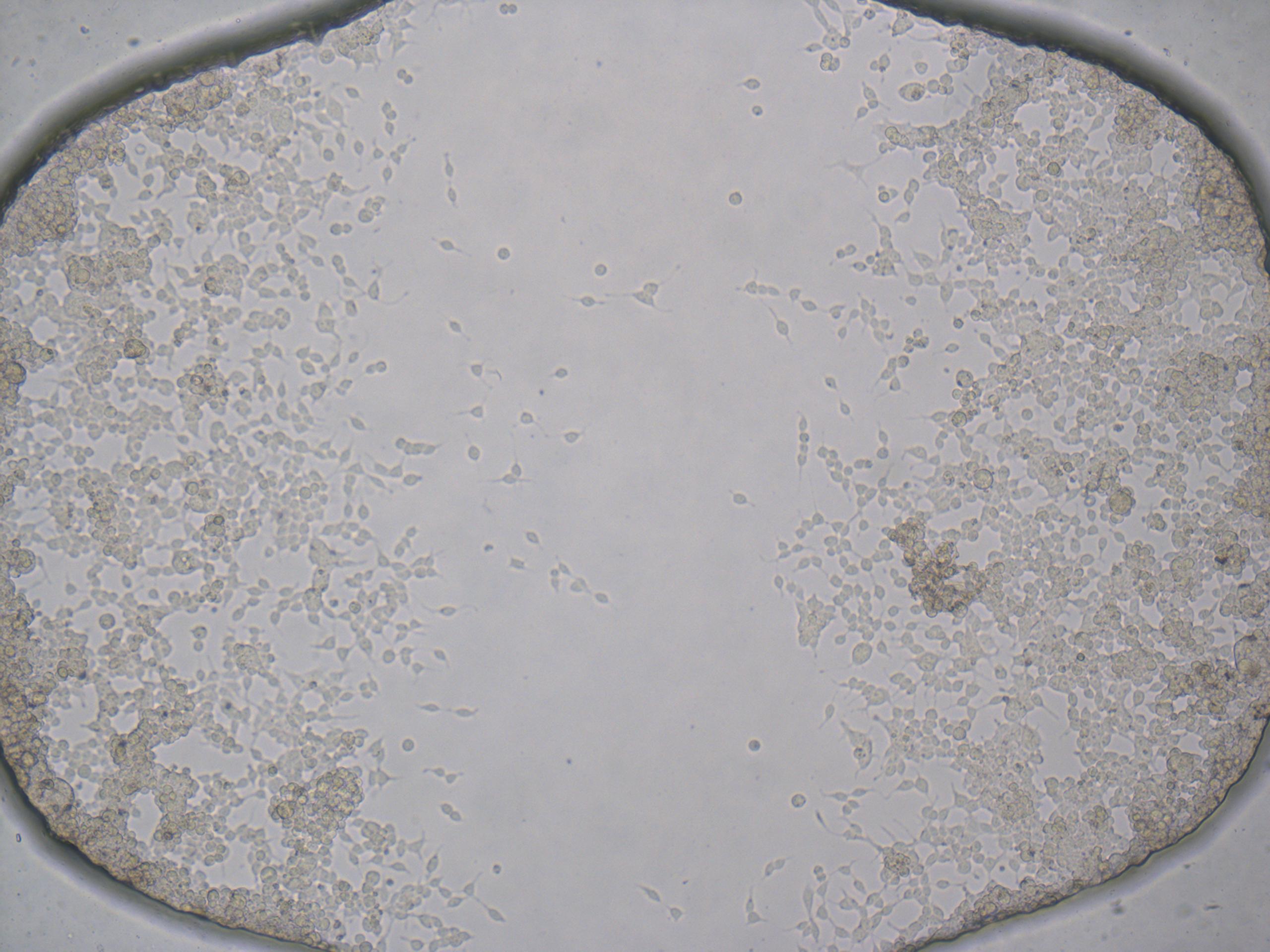

Supplement: S1 File — (ZIP) [file pone.0193605.s001.zip › source_code/input/7/2.5/2016-11-25-G2-8e5hek.jpg]

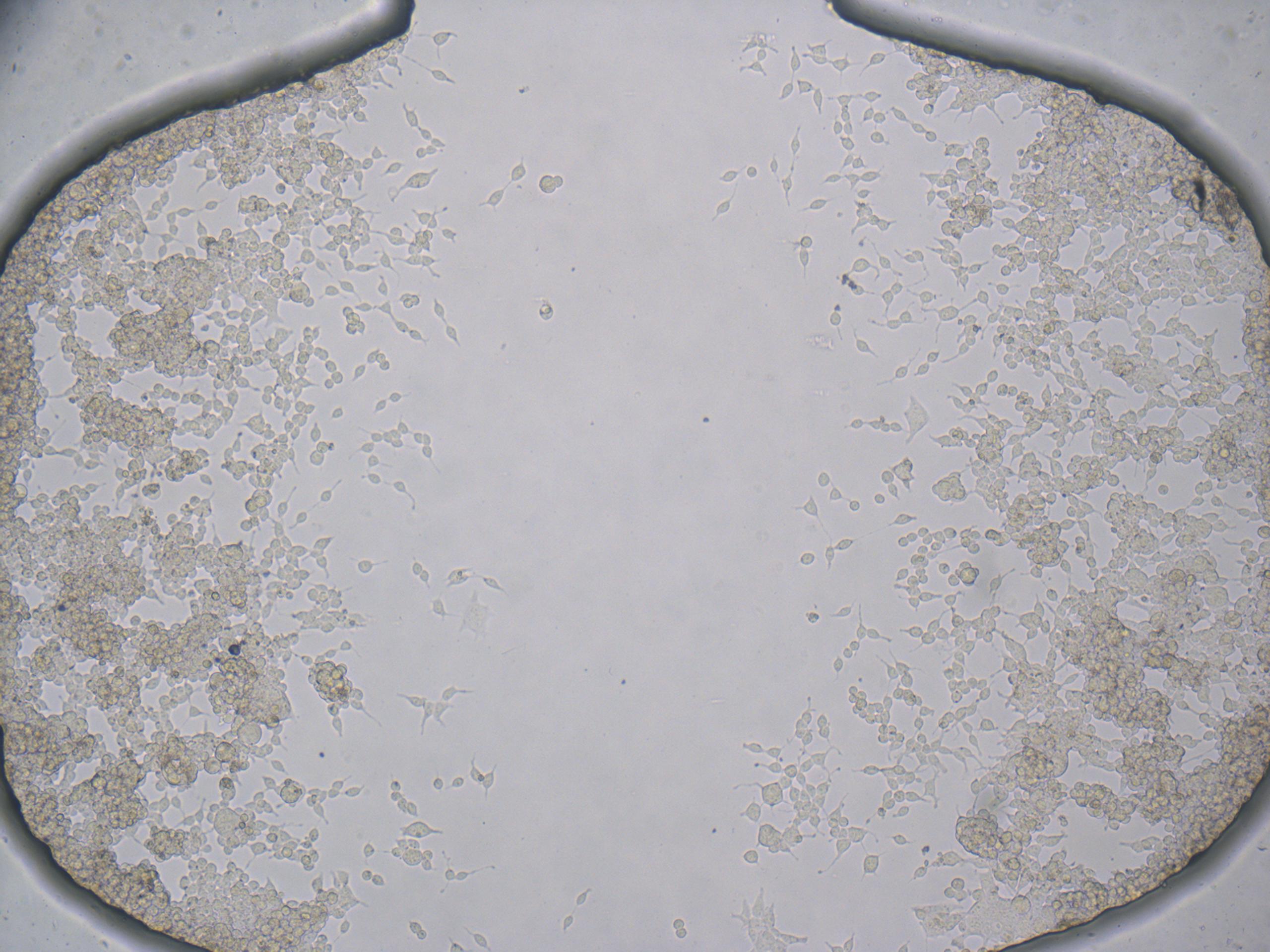

Supplement: S1 File — (ZIP) [file pone.0193605.s001.zip › source_code/input/7/2.5/2016-11-25-G4-8e5hek.jpg]
